# Early multiple sclerosis activity associated with TBX21^+^CD21^lo^CXCR3^+^ B cell expansion resembling EBV-induced phenotypes

**DOI:** 10.1172/jci.insight.188543

**Published:** 2025-05-13

**Authors:** Elliott D. SoRelle, Ellora Haukenfrers, Gillian Q. Horn, Vaibhav Jain, James Giarraputo, Karen Abramson, Emily Hocke, Laura A. Cooney, Kristina M. Harris, Scott S. Zamvil, Simon G. Gregory, Micah A. Luftig

**Affiliations:** 1 Department of Molecular Genetics and Microbiology, Duke University School of Medicine, Durham, North Carolina, USA.; 2 Duke Molecular Physiology Institute, Duke University, Durham, North Carolina, USA.; 3 Department of Integrative Immunobiology, Duke University School of Medicine, Durham, North Carolina, USA.; 4 Yale School of Medicine, New Haven, Connecticut, USA.; 5 Immune Tolerance Network, Bethesda, Maryland, USA.; 6 Department of Internal Medicine, Division of Rheumatology, University of Michigan, Ann Arbor, Michigan, USA.; 7 Department of Neurology, University of California, San Francisco, San Francisco, California, USA.; 8 Department of Neurology, Duke University School of Medicine, Durham, North Carolina, USA.

**Keywords:** Autoimmunity, Immunology, Virology, Autoimmune diseases, Multiple sclerosis

## Abstract

Epstein-Barr virus (EBV) infection precedes multiple sclerosis (MS) onset and plays a poorly understood etiologic role. To investigate possible viral pathogenesis, we analyzed single-cell expression in peripheral B cells from people with early MS collected longitudinally during the Immune Tolerance Network STAyCIS Trial. Expression profiles were compared with single-cell RNA-Seq (scRNA-Seq) from in vitro EBV models, autoimmune disorders, chronic infectious diseases, and healthy controls. Analyses focused on CD19^+^CD20^+^CD21^lo^CD11c^+^T-bet^+^ atypical B cells (ABCs). ABCs were significantly enriched in early MS PBMCs versus healthy controls by scRNA-Seq and flow cytometry, establishing ABC expansion as a clinical feature. EBV-associated ABC expression, including CXCR3, programmed cell death ligand 1 (PD-L1), and PD-L2, was enriched in early MS; however, direct EBV infection of ABCs was not detected. Early MS ABCs exhibited significantly upregulated inflammatory cytokine mRNAs (*CXCL8*, *IL18*, *VEGFA*). Further, de novo EBV-infected B cells secreted IL-8 and VEGF. MS activity stratification revealed rare, distinctive inflammatory ABCs significantly underrepresented in individuals with no evidence of activity long-term versus people with additional relapsing-remitting MS activity at the primary endpoint. Moreover, CXCR3^+^ ABCs increased after baseline diagnosis and were significantly enriched in people with disease exacerbation during the study. Thus, ABC expansion and inflammatory responses correlate to early MS activity, possibly as a bystander response to EBV.

## Abstract 2

Longitudinal single-cell analyses of B cells from people with early MS identify peripheral enrichment of atypical B cells expressing CXCR3, immune checkpoint molecules, and an EBV-associated pro-inflammatory phenotype linked with subsequent disease activity.

## Graphical abstract

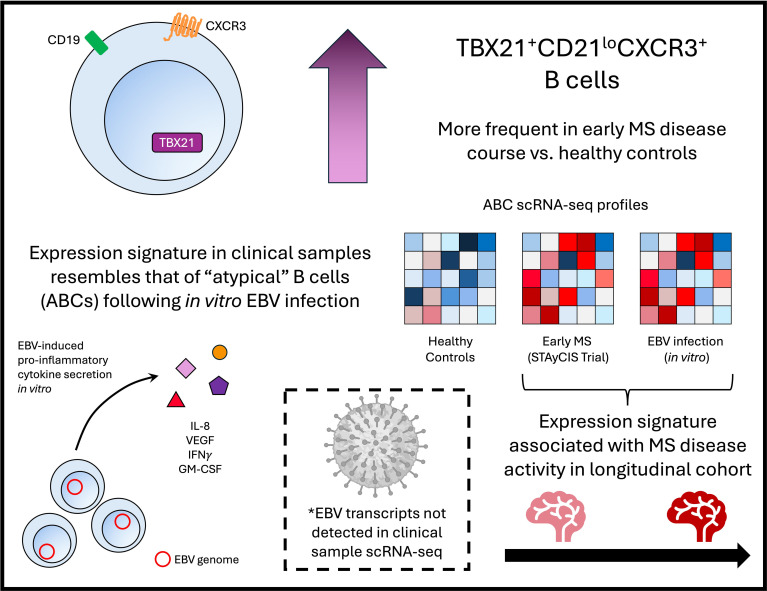

## Introduction

The atypical B cell (ABC) compartment comprises immune cells coexpressing the T cell lineage transcription factor T-bet (*TBX21*), the plasmacytoid dendritic cell marker CD11c (*ITGAX*), and the classical B cell markers CD19 and CD20 (*MS4A1*) but lacking CD21, CD27, and IgD (1, 2). Originally discovered in mice as age associated, these cells accumulate in response to Toll-like receptor 7 (TLR7) and interferon-γ (IFN-γ) stimulation and exhibit B cell receptor anergy in addition to molecular indicators of exhaustion and germinal center–independent (GC-independent) development (1, 3–7). ABC pools contain high frequencies of autoreactive clones and are expanded in females due at least partially to dosage effects of TLR7, which is encoded on the X chromosome and escapes X-linked inactivation (1, 8–11). Although present in healthy individuals and capable of mediating protective immune responses (12–14), T-bet^+^ B cells coexpressing combinations of other markers (e.g., Fc receptor like 5 [FCRL5]/CXCR3) are commonly pathogenic activated effectors in autoimmune and infectious diseases with female bias (15, 16). These include systemic lupus erythematosus (SLE) (9, 17–19), primary Sjögren’s syndrome (PSS) (20), rheumatoid arthritis (21, 22), multiple sclerosis (MS) (23), and chronic infections with HIV and malaria (MAL) (24–29).

Epstein-Barr virus (EBV) is a gammaherpesvirus establishing lifelong infection in more than 90% of adults worldwide (30). This prevalence is attributable to the success with which EBV induces and modulates adaptive immune programs of host B cells to achieve persistent latency in the memory B cell pool via GC-dependent and GC-independent routes (31–34). Beyond its carcinogenic potential (35), EBV is frequently associated with autoimmune and chronic infectious diseases that exhibit clonally expanded ABCs (36–47). We recently reported that EBV can infect existing ABCs (34) and promote ABC generation in vitro (48). However, the manifestation and mechanistic contributions of EBV infection in autoimmune and chronic infectious diseases remain incompletely understood.

MS is a chronic neuroinflammatory disease with a 3:1 female bias (23) in which both pathogenic ABCs (49, 50) and epidemiologic evidence of EBV etiology in disease onset (a 32-fold risk from EBV seropositivity after childhood) have been identified (51, 52). Several hypotheses have been proposed to explain EBV involvement in MS: immune responses to viral proteins cross-reactive with self-antigens (molecular mimicry), EBV-induced licensing of forbidden autoreactive B cell clones, and inflammatory damage from infected cells (53–55). Antibody reactivity and T cell profiles from people with MS show molecular mimicry between the viral latency protein EBV nuclear antigen 1 (EBNA1) and CNS-expressed host proteins (56, 57). Multiple EBV antigens and EBV^+^ B cells have been detected in MS brain lesions though inconsistently (58–63). Notably, a correlation between neuroinvasive CXCR3^+^ B cells and EBV viral load exists in patients with MS (64). Beyond shedding light on the clinical efficacy of B cell depletion therapy, these findings underscore immune dysregulation mediated by EBV in B cells within the CNS. How the virus accesses the CNS and promotes pathogenesis remains unclear, but clinical aspects of MS align with markers and functions of ABCs, suggesting EBV infection perturbs this immune niche.

This study applies single-cell sequencing to investigate B cells from multiple diseases, focusing on samples from the STAyCIS Trial (65), which studied atorvastatin efficacy in preventing relapses or new CNS lesions in individuals diagnosed with clinically isolated syndrome (CIS) per Controlled High-Risk Subjects Avonex Multiple Sclerosis Prevention Study criteria that led to the approval of intramuscular IFN-β1a (66). Patients diagnosed with MS by 2001 McDonald criteria were excluded (67), though CIS at the time would likely be diagnosed as early MS by current criteria; we use the terminology early MS (eMS) herein. CIS is an initial clinical episode of neurologic symptoms caused by neuroinflammation and demyelination often preceding MS (68). People with CIS presenting with brain or spinal cord lesions detected by MRI have 60%–80% chance of subsequent neurologic events and relapsing-remitting MS (RRMS) diagnosis (69–71). However, ABC characteristics in CIS (here, eMS) cohorts remain understudied, as does whether signatures of ABC response to EBV infection exist in eMS or across virus-associated diseases (34). To address these gaps, we analyzed ABCs from people with eMS and compared them with ABCs from other disease cohorts and in vitro EBV infection.

## Results

### Study population and procedures.

Peripheral blood mononuclear cells (PBMCs) were collected from a subset of the original Immune Tolerance Network (ITN) STAyCIS Trial cohort of people (*n* = 16 of 81 original participants; 11 female, 5 male) at 2 time points following initial diagnosis: t_1_ (baseline visit; at least 28 days after completion of corticosteroid treatment and within 210 days of presentation) and t_2_ (at least 3 months post-t_1_; mean = 172 days post-t_1_, range = 84–357 days). Beginning at t_1_, 11 people were treated with atorvastatin and 5 were given a placebo as part of the ITN STAyCIS Trial (65) (ClinicalTrials.gov NCT00094172). Treatments were given for 12 months or until participants met the primary endpoint of MS activity, defined as 3 or more new T2 lesions or 1 clinical exacerbation. Eight of the 16 participants for whom samples were analyzed met the primary endpoint within the 12-month treatment window, and 2 additional participants met the primary endpoint in a follow-up period between 12 and 18 months. Of the remaining 6 participants, 3 were subsequently diagnosed with MS based on new T2 lesions. Thus, 13 of 16 participants in the analyzed cohort were diagnosed with MS based on the accepted McDonald criteria at that time. The remaining 3 participants were characterized post hoc as long-term no evidence of activity (LTNA) based on the collective absence of new T2 lesions, Gd-enhancing lesions, and clinical exacerbations in the 18 months following t_1_. Of the 13 individuals with additional after-baseline MS activity, 10 received atorvastatin and 3 received placebo; of the 3 LTNA individuals, 2 received atorvastatin and 1 received placebo. In the original trial, atorvastatin significantly reduced development of new T2 lesion activity but did not meet the combined imaging and clinical primary endpoint (65). Notwithstanding, the longitudinal cohort provides a unique window into the peripheral lymphocyte compartment in eMS stages.

**Figure 1:**
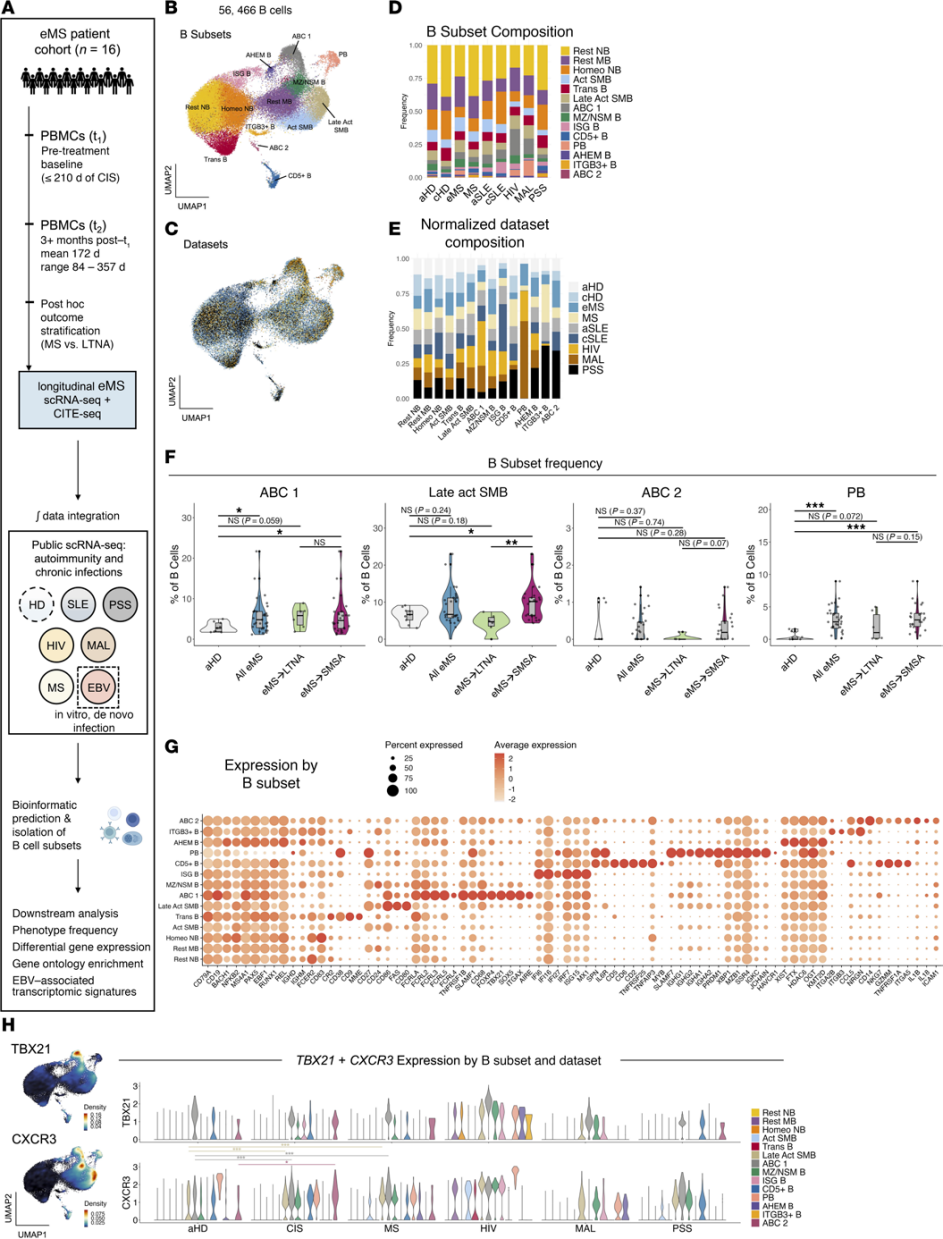
ABCs are enriched in people with eMS who experience subsequent MS activity. (**A**) Overview of sample collection, analysis workflow, and dataset integration from eMS cohort with publicly available data. (**B**) Integrated uniform manifold approximation and projection (UMAP) by B cell subsets. Rest NB, resting naive B cells (yellow); Rest MB, resting memory B cells (light purple); Homeo NB, homeostatic naive B cells (orange); Act SMB, activated switched memory B cells (powder blue); Trans B, transitional B cells (red); Late Act SMB, late activated switched memory B cells (beige); ABC 1, atypical B cell phenotype 1 (dark gray); MZ/NSM B, marginal zone/non-switched memory B cells (green); ISG B, interferon-stimulated gene signature B cells (pink); CD5+ B, CD5^+^ B cells (blue); PB, plasmablasts (peach); AHEM B, B cells with upregulated autophagy, ATP hydrolysis, and epigenetic modifier expression (dark purple); ITGB3+ B, ITGB3^+^ITGA2B^+^ B cells (goldenrod); ABC 2, atypical B cell phenotype 2 (fuchsia). (**C**) Integrated UMAP by dataset. aHD, adult healthy donor (eggshell white); cHD, child healthy donor (light blue); eMS, early MS (medium blue); MS, multiple sclerosis (cream); aSLE, adult systemic lupus erythematosus (gray); cSLE, child systemic lupus erythematosus (dark blue); HIV, chronic HIV infection (gold); MAL, chronic malaria (bronze); PSS, primary Sjögren’s Syndrome (black). (**D**) B cell subset composition across conditions. (**E**) B cell subset composition by condition, normalized by dataset sample size. (**F**) Statistical comparisons of B subset frequencies in aHDs, all eMS, and eMS stratified by LTNA (seafoam green) and SMSA (magenta) outcomes. Two-sided Wilcoxon rank-sum test (*P < 0.05; **P < 0.01; ***P < 0.001). (**G**) Scaled expression of B subset marker genes. (**H**) TBX21 and CXCR3 expression.

PBMCs from each individual and time point were prepared as single-cell libraries (single-cell RNA-Seq [scRNA-Seq] + cellular indexing of transcriptomes and epitopes by sequencing [CITE-Seq]), which were sequenced, quality-controlled, and aligned to generate count matrices. Single-cell data from the eMS cohort were then analyzed in conjunction with publicly available scRNA-Seq datasets from autoimmune disorders (MS, SLE, PSS) (49, 72–74) and chronic infectious diseases (HIV, MAL) (27) in which pathogenic ABCs have been described, as well as healthy controls (Figure 1A). Peripheral B cells (naive, intermediate, memory, and plasmablasts) were identified from each dataset by cell type prediction with Azimuth (75) and isolated for downstream analysis. We also incorporated the analysis of ABCs prior and subsequent to EBV infection in vitro (34) to assess transcriptomic similarities among ABCs from disease and in vitro EBV infection. The eMS dataset contained 14,969 annotated B cells collected from individuals across the 2 time points (*n* = 32). Total B cells and library sizes for all datasets are provided as supplemental material (Supplemental Table 1; supplemental material available online with this article; https://doi.org/10.1172/jci.insight.188543DS1). B cells from the eMS cohort and publicly available datasets were integrated using single-cell batch correction methods (75) into a single object containing about 57,000 cells. Unbiased clustering was applied to refine high-resolution B subsets via biomarker analysis and literature-based annotation (Figure 1B). Analysis of integrated B cells by dataset verified successful correction of batch and sequence depth variation (Figure 1C and Supplemental Figures 1 and 2).

### Characterization of B cell populations within study cohort compared with chronic infection, autoimmunity, and healthy controls.

Percentages of B cells within annotated subsets by disease were assessed (Figure 1D), verifying findings in prior studies such as ABC expansion (ABC 1) in SLE, HIV, and MAL; B cells with ISG signature (ISG B) in SLE; and PB enrichment in HIV and MAL. Generally, B cell compartments in chronic infections and autoimmune diseases were skewed toward lower frequencies of resting naive and memory B cells and higher frequencies of activated effector phenotypes (e.g., activated switched memory B cells, PBs). The proportion of cells from each disease context and healthy controls for each annotated B subset was quantified after normalizing for total cell numbers per dataset (Figure 1E). This representation revealed enrichment of comparatively rare B cell phenotypes in disease versus healthy controls. Beyond PB predominance from HIV, MAL, and eMS, we identified overrepresentation of eMS- and PSS-derived B cells with gene ontology (GO) enrichment for AHEM B; MS- and PSS-derived ITGB3^+^ B; and eMS- and PSS-derived ABC 2, an apparent, distinct, rare population of ABCs (based on coexpression of *TBX21*, *CD19*, *MS4A1*, and *ITGAX*). Statistical comparisons identified significant enrichment of ABC 1 (2-sided Wilcoxon rank-sum test, *P* = 0.031) and PB (Wilcoxon *P* = 1.55 × 10^–4^) in eMS versus healthy adults. Conversely, B cells distinguished by elevated expression of CD5 (CD5^+^ B) were less frequent in patients with eMS versus healthy controls (Wilcoxon *P* = 2.71 × 10^–3^). Stratification of the eMS cohort by subsequent MS activity (eMS→SMSA) versus individuals with no additional evidence of MS activity (eMS→LTNA) revealed differences in frequencies of late activated switched memory B cells (Late Act SMB; aHD < eMS→SMSA, Wilcoxon *P* = 0.042; eMS→LTNA < eMS→SMSA, Wilcoxon *P* = 2.72 × 10^–3^) and AHEM B cells (aHD < eMS→SMSA, Wilcoxon *P* = 0.036). Samples from eMS→SMSA displayed elevated ABC 2 frequencies relative to eMS→LTNA samples, though not statistically significant (Wilcoxon *P* = 0.07) (Figure 1F and Supplemental Figure 3A). Notably, no significant differences in B subset frequencies existed between atorvastatin versus placebo groups (Supplemental Figure 3B).

Next, we assessed differential gene expression for each annotated subset across integrated B cell datasets (Figure 1G and Supplemental Tables 2 and 3). Conventional naive and memory B subsets (Rest NB, Rest MB, Homeo NB) were identified by patterns of *CD19*, *FCER2* (*CD23*), *CD24*, *CD27*, CD38, *IGHD*, *IGHG1*, and other B cell lineage markers. Trans B cells were distinguished by elevated *CD9* and *MME* (*CD10*) expression. Activated B subsets were further delineated by expression of *CD86*, *FAS* (*CD95*), and *CD80*. In addition to elevated expression of *TBX21* (T-bet), *ITGAX* (CD11c), and *FCRL5*, ABC 1 cells were distinguished by *FOXP4*, *SOX5*, *TNFRSF1B*, near-exclusive expression of *AIRE*, and low expression of *CR2* (CD21). ISG B cells displayed the highest levels of *IFI6*, *IFI16*, *IFI27*, and *ISG15*. PBs expressed high *SPN* (*CD43*), *SLAMF7*, *PRDM1* (*BLIMP-1*), and *SDC1* (*CD138*). The CD5^+^ B phenotype also expressed *CD6*, *CD2*, *MYB*, and *TNFRSF25*, while ITGB3^+^ B cells expressed *ITGA2B* and moderate levels of *CD9*. AHEM B cells displayed elevated mitochondrial gene expression, sex-specific expression of *XIST* and its regulator *FTX*, and the epigenetic regulators *HDAC6* and *KMT2D*. Finally, the ABC 2 cluster expressed inflammatory cytokine transcripts (*IL1B* and *IL18*) in addition to atypical B lineage genes (Figure 1G and Supplemental Figure 4). An examination of *TBX21* and *CXCR3* by B subset and dataset revealed that these genes were generally expressed at higher levels and in more B subsets in disease contexts versus healthy adults. Specifically, *CXCR3* expression in ABC 1 and Late Act SMB subsets was significantly higher in eMS and MS samples versus healthy controls (Figure 1H). ABC 2 expression of *CXCR3* and *TBX21* was also elevated in eMS versus healthy controls, though only *CXCR3* enrichment was significant by statistical test (*CXCR3* Wilcoxon *P* = 0.043; *TBX21* Wilcoxon *P* = 0.11) (Figure 1H). Variation in *CXCR3* expression across diseases may reflect differential enrichment of B cells originating from distinct cell lineages (76) or changes in the balance of resting (*CXCR3*^–*/*lo^) versus stimulated (*CXCR3*^hi^) phenotypes (48, 77, 78). Collectively, scRNA-Seq resolved peripheral B cell landscape variations in health and disease and defined the expansion of *CXCR3*^+^ B subsets, including *TBX21*^+^CD21^lo^ ABCs, as clinical features in people with eMS.

### EBV-induced gene expression signature is enriched in eMS patient ABCs.

**Figure 2:**
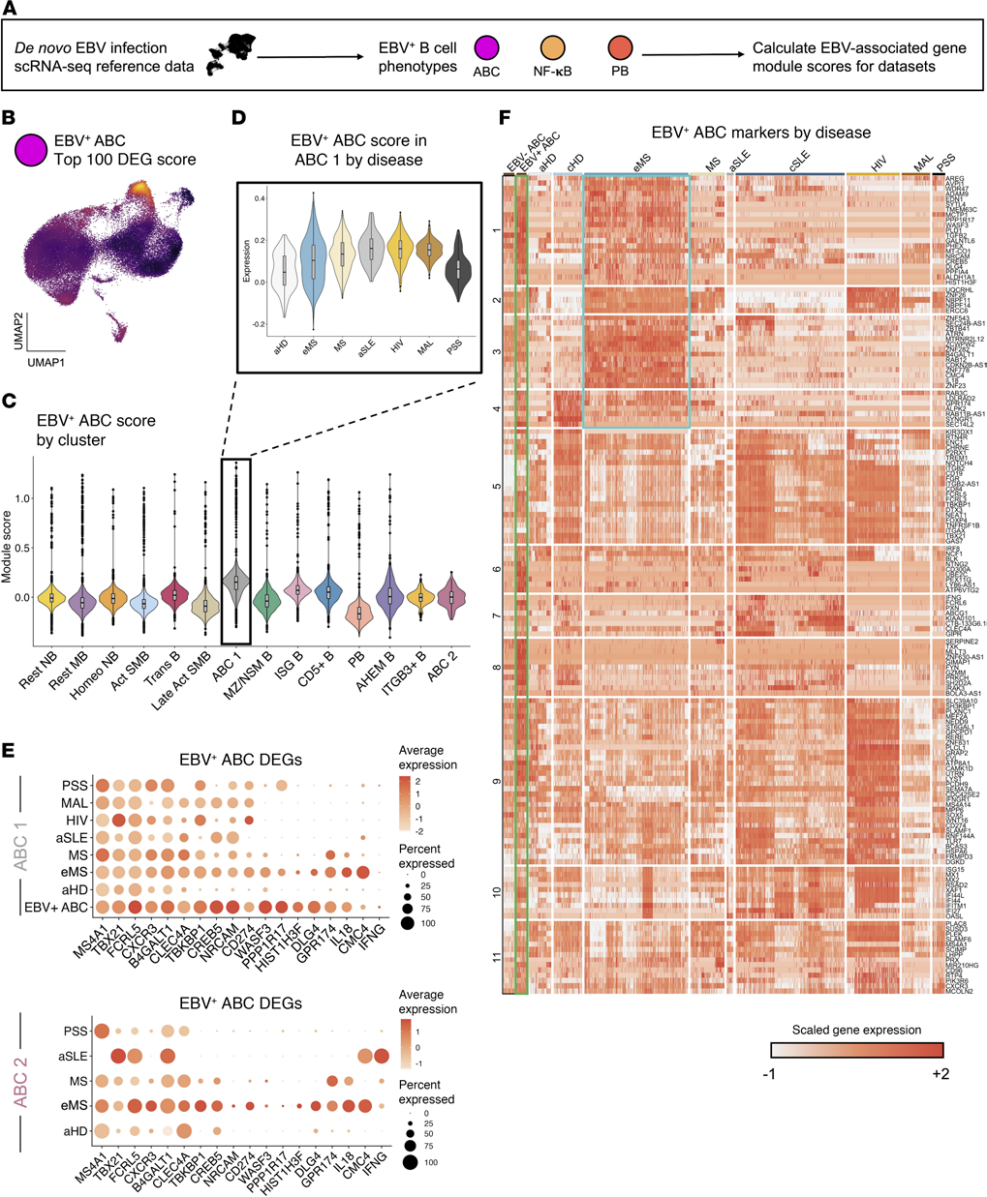
EBV-associated gene expression signatures by B subset, disease, and clinical outcome. (**A**) Strategy to assess single-cell gene expression signatures associated with de novo EBV infection of B cells in vitro. ABC, EBV^+^ atypical B cell; NFκB, EBV^+^ NF-κB activated B cell phenotype; PB, EBV^+^ plasmablast phenotype. (**B**) UMAP representation of the in vitro EBV^+^ ABC gene signature across all integrated B cells from disease and healthy control datasets. DEGs, differentially expressed genes. (**C**) EBV^+^ ABC signature module scores by B cell state. Scores were calculated from the top 100 DEGs in EBV^+^ ABCs versus resting ABCs in vitro. (**D**) EBV^+^ ABC signature module scores in the ABC 1 cluster stratified by disease and healthy control datasets. (**E**) Dot plots of EBV^+^ ABC biomarker expression across ABC 1 (top panel) and ABC 2 (bottom panel) subsets in disease cohorts and healthy adults. (**F**) EBV^+^ ABC biomarker expression by dataset. Single-cell expression heatmap of ABC biomarkers and EBV-induced genes in ABCs during in vitro early infection. Genes (rows) are clustered by expression pattern similarity across datasets. Resting (EBV^–^) and infected (EBV^+^) ABCs from in vitro experiments are outlined in black and green boxes, respectively. EBV^+^ ABC gene groups identified by unbiased hierarchical ordering to have elevated expression in ABCs from patients with eMS but not healthy adults are outlined in cyan.

Based on the positive association between CNS-infiltrating CXCR3^+^ B cells and EBV viral load in people with MS (64) and our prior identification of EBV-induced CXCR3 expression (34, 48), we next evaluated expression of EBV-induced gene expression signatures within the integrated B cell scRNA-Seq data (Supplemental Table 4, Figure 2, and Supplemental Figures 5–9). Using scRNA-Seq from in vitro EBV infection, we generated the top 100 DEGs in distinct EBV-induced phenotypes, including a comparison of EBV^+^ versus EBV^–^ ABCs (Figure 2A and Supplemental Figure 5, A–C). The EBV^+^ ABC module score was highest in the ABC 1 subset as expected, with elevated scores observed for ABCs from all autoimmune diseases and chronic infections relative to healthy control ABCs (Figure 2, B–D). The EBV^+^ NF-κB activation signature was high in multiple activated effector subsets and strongest in Late Act SMB cells, though this module did not exhibit clear patterns between healthy and disease contexts (Supplemental Figure 5A). The PB gene module and curated ISG signature were elevated, respectively, in PB and ISG B subsets as expected, with higher ISG scores observed in eMS, SLE, HIV, and PSS (but not MS or MAL) relative to healthy adults (Supplemental Figure 5, B and C, and Supplemental Figure 6). Although GO results indicated similar biological process enrichment in Late Act SMBs and ABCs in eMS (Supplemental Figure 7), we focused our analysis on the 2 ABC subsets. One of these (ABC 1) was more frequent in people with eMS regardless of disease outcome and time point, while the other (ABC 2) was elevated in people with subsequent MS activity (eMS→SMSA). ABC 1 and ABC 2 subsets from people with eMS exhibited higher scores for genes in the top 100 EBV^+^ ABC DEGs when compared with ABCs from healthy donors (Supplemental Tables 5 and 6). These included *CXCR3*, *B4GALT1*, *CLEC4A*, *TBKBP1*, *CREB5*, *NRCAM*, *CD274* programmed cell death ligand 1 (PD-L1), *WASF3*, *PPP1R17*, *HIST1H3F*, *DLG4*, and *GPR174*. Other EBV^+^ ABC markers (not within the top 100 DEGs) elevated in one or both eMS ABC subsets versus healthy donors included *IL18*, *CMC4*, and *IFNG*. Several of these EBV-associated genes expressed in ABCs were also upregulated in other disease contexts (Figure 2E and Supplemental Figure 8).

Hierarchical clustering of ABC lineage and EBV^+^ ABC genes refined single-cell expression patterns in ABC subsets by disease status (Figure 2F and Supplemental Figure 9). Genes including *CD84*, *ENC1*, *FCGR2A* (CD32), the EBV EBNA2 target *FGR* (79, 80), *FOXP4*, *GAS7*, *GRAP2*, *HCK*, *MPP6*, *PLEK*, and *TNFRSF1B* were consistently expressed by ABCs in most contexts, while certain genes were restricted to ABC clusters in one or more diseases but not healthy controls. Based on their functional importance to ABC responses, we examined genes with annotated roles in IFN-γ and TLR7 signaling. Indeed, *FCRL3*, *PIK3AP1*, *SCIMP*, and *SLAMF6* were upregulated across ABCs. *TLR7* expression, which is known to be induced upon EBV virion entry into B cells (81), was upregulated in ABCs from HIV, SLE, eMS, and MS versus healthy adults. Collectively, this analysis supported prior findings of ABC pathogenicity in autoimmunity and chronic infection, possibly mediated by common mechanisms (27, 82). The presence and extent of shared expression trends between disease state and EBV-infected ABCs further supported a potential association between EBV infection and pathogenic ABC expansion.

Expression of other EBV^+^ ABC genes including *ALDH1A1*, *AREG*, *EDN1*, *SYTL4*, *TMEM63C*, *MCTP1*, *PPP1R17*, and *PRX* was mostly specific to eMS and a subset of MS ABCs. Many of the EBV^+^ ABC genes not expressed in healthy controls play roles in development, cell plasticity, or neuronal lineage specification and were infrequently expressed in mature B subsets. It was striking that an idiosyncratic collection of genes upregulated in ABCs following EBV infection was also observed in disease-associated ABCs, but not healthy controls, and that these transcriptomic similarities were greatest in eMS. The lineage-ectopic nature of this signature may be due to unknown or uncharacterized functional roles for these gene products in B cells. Alternatively, it is conceivable that their expression, not normally detected in B cells, may be associated with induced reprogramming that promotes or maintains cellular plasticity (83–86). In this regard, we speculate that ABCs may be primed for aberrant responses to complex stimuli including viral infection.

### EBV-regulated genes are commonly expressed in eMS/MS ABCs relative to healthy controls.

**Figure 3:**
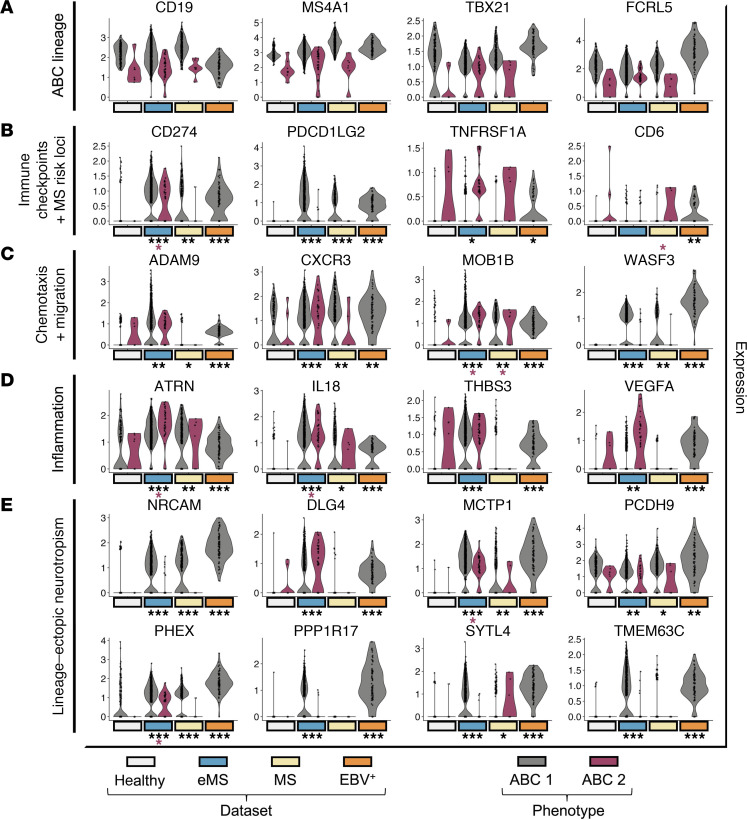
EBV-induced ABC markers observed in eMS ABCs are associated with pathogenic immune checkpoint evasion, neuroinvasive chemotaxis, and inflammatory responses. (**A**) ABC lineage marker expression within peripheral ABC 1 (gray) and ABC 2 (fuchsia) subsets across healthy adults (eggshell white), people with eMS (medium blue), people with MS (beige), and in vitro EBV infection (orange). (**B**) EBV^+^ ABC biomarkers: immune checkpoint and MS susceptibility gene expression color-coded by ABC phenotypes and dataset as in **A**. Statistically significant differences relative to healthy controls are represented by gray asterisks (ABC 1 phenotype) and fuchsia asterisks (ABC 2 phenotype) (Kolmogorov-Smirnov [KS] test, *P < 0.05; **P < 1 × 10^–5^; ***P < 1 × 10^–10^). (**C**) EBV^+^ ABC biomarkers: chemotactic and cell migration gene expression color-coded by ABC phenotypes and dataset as in **A** and **B**. Statistically significant differences in expression relative to healthy controls are represented as in **B**. (**D**) EBV^+^ ABC biomarkers: inflammatory mediator expression color-coded by ABC phenotypes and dataset as in **A**–**C**. Statistically significant differences in expression relative to healthy controls are represented as in **B** and **C**. (**E**) EBV^+^ ABC biomarkers: expression of lineage-ectopic neurotropic genes color-coded by ABC phenotypes and dataset as in **A**–**D**. Statistically significant differences in expression relative to healthy controls are represented as in **B**–**D**.

Given these observations, we further examined the possibility of EBV-associated ABC pathogenicity (Figure 3 and Supplemental Figure 9B). ABC lineage markers, including *CD19*, *MS4A1* (CD20), *TBX21* (T-bet), and *FCRL5*, were conserved at similar levels in each ABC subset from healthy adults, eMS, MS, and in vitro EBV infection (Figure 3A). By contrast, significant upregulation of the immune checkpoint genes *CD274* (PD-L1) and *PDCD1LG2* (PD-L2) was observed in eMS, MS, and de novo EBV-infected ABCs versus healthy controls. The EBV^+^ ABC marker and MS susceptibility gene *TNFRSF1A* was significantly elevated in ABC 1 from people with eMS versus healthy adults (Wilcoxon *P* = 7.99 × 10^–7^). *CD6* (another MS risk locus observed in EBV^+^ ABCs) was mildly elevated in ABC 2 from eMS, but not significantly (Wilcoxon *P* = 0.19), versus healthy controls (Figure 3B). Several genes upregulated in EBV^+^ and eMS ABCs are mediators of chemotaxis and tissue invasion, including the metalloproteinase *ADAM9*, which is induced in peripheral and tissue-infiltrating mononuclear cells under inflammatory conditions (87). While *CXCR3* was expressed in ABC 1 from healthy donors, it was significantly upregulated in ABC 1 cells from people with eMS (Wilcoxon *P* = 4.58 × 10^–9^) and MS (Wilcoxon *P* = 4.6 × 10^–14^) and in ABC 2 cells from people with eMS (Wilcoxon *P* = 0.043). *CXCR3* is notable because it facilitates migration from lymph nodes to inflammatory sites (88, 89) and has been defined as a characteristic of neuroinvasive B cells in diseases including MS (64, 90, 91). Because CXCR3^+^ B cell frequency in MS is positively correlated with EBV viral load (64) and the virus can induce B cell CXCR3 mRNA and protein expression (34, 48), infection may promote ABC trafficking to the CNS. *WASF3*, another enriched gene in ABC 1 from eMS and MS, encodes an actin-binding protein that regulates cytoskeletal morphology and promotes cellular metastasis and invasion in breast and prostate cancer (92, 93) (Figure 3C). Enrichment of numerous pro-inflammatory mediators (*IL18*, *THBS3*, *VEGFA*) associated with de novo EBV infection was also observed in ABC subsets from people with eMS (Figure 3D). In particular, ABC 2 cells were distinguished by elevated expression of genes encoding chemokine ligands, cytokines, and secreted factors with pro-inflammatory effects (Supplemental Figure 9B). These included CC motif chemokine ligands *CCL3* (MIP-1A), *CCL4* (MIP-1B), *CCL5* (RANTES); CXC motif chemokine ligands *CXCL2* (MIP-2A), *CXCL3* (MIP-2B), *CXCL8* (IL-8); granzymes *GZMK*, *GZMM*; *IFNG* (IFN-γ); *IL18*; *IL1B*; and *TNF* (TNF-α) — several of these have been previously identified in people with MS (94–96). *CCL3*, *CCL4*, *CCL5*, and *IL18* were also significantly upregulated at the mRNA level during de novo EBV infection of ABCs. Elevated expression of *IL18* and the pro-fibrotic wound-healing mediators *AREG* (97) and *PDGFD* (98) within EBV^+^ and eMS phenotypes further indicated potential functions or participation in (neuro)inflammatory responses. In this regard, expression of neuronal genes involved in cell-cell interactions and axon guidance (e.g., *NRCAM*, *MCTP1*, *PPP1R17*, *SYTL4*, *TMEM63C*) was further suggestive of neurotropism (Figure 3E). Upregulation of *DLG4* in EBV^+^ and eMS ABCs was noteworthy, as this gene encodes a neuronal signaling mediator identified in MS leukocytes that was significantly downregulated during IFN-β treatment (99).

To investigate potential contributions of EBV infection to cytokine production and inflammatory responses, we performed a 27-plex Luminex assay comparing secretion profiles of resting peripheral B cells, early EBV-infected cells, and EBV-immortalized lymphoblastoid cell lines (LCLs). Secreted CCL2, CCL3, CCL4, CCL5, CXCL10, GM-CSF, IFN-γ, IL-8, IL-10, IL-1RA, TNF-α, and VEGF were all significantly elevated in de novo EBV-infected proliferative B cells relative to resting cells (2-sided Wilcoxon rank-sum tests, *P* < 0.05 for all). Secreted CCL3, CCL4, CCL5, CXCL10, IFN-γ, IL-10, and VEGF were also significantly elevated in LCLs but at lower levels than in de novo infection (Supplemental Figure 10).

We next applied GO enrichment to compare ABC 1 cells from outcome-stratified eMS samples versus healthy adults (Supplemental Figure 11, A and B). Regardless of outcome, top enriched terms for ABC 1 in the eMS cohort versus ABC 1 in healthy adults were related to viral transcription and mRNA catabolism, including nonsense-mediated decay, which mediates antiviral immunity in RNA and DNA virus infections (100–102). Despite these findings, we did not directly detect EBV viral transcripts in any B subset from people with eMS via read alignment against a multispecies reference genome. Thus, it cannot be definitively determined whether pathogenic features of ABC subsets in people with eMS detailed above are directly induced by EBV infection or represent ABC-specific responses that may be triggered indirectly by EBV infection or other stimuli.

### ABC gene expression programs correlate with subsequent MS activity after baseline.

We next extensively characterized differences in B cell composition, phenotypes, and ABC compartments between eMS→SMSA and eMS→LTNA groups. After accounting for different sample sizes, resting or homeostatic naive B cells (Rest NB, Homeo NB) were more frequent in eMS→LTNA samples. The ABC 1 subset was present at similar frequencies in SMSA and LTNA outcomes. The ISG B subset was more prevalent in the LTNA group; however, this was attributable to an individual sample. The ABC 2 phenotype was overrepresented in people with eMS who experienced subsequent MS disease activity (Supplemental Figure 12A). Cell-matched surface protein expression assayed via CITE-Seq verified that ABC 1 and ABC 2 subsets were CD19^+^CD11c^+^CD27^lo^ (Supplemental Figure 12, B and C). Further, CD19 and CD11c expression were elevated in ABCs from eMS→SMSA samples. Intriguingly, ABC 2 cells (*n* = 37 cells, 36 of which were derived from eMS→SMSA outcomes) displayed broad immune lineage expression including CD11b (ITGAM), CD14, and CD64 (Supplemental Figure 12C). Likewise, ABC 2 cells exhibited an unusually broad set of surface-expressed genes in the scRNA-Seq assay, many of which were expressed at higher levels in eMS and MS versus healthy controls (Supplemental Figure 11C). The most distinctive of these included *CD14*, *CD33*, *CD274* (PD-L1), *HAVCR2* (CD366), *ITGAM* (CD11b), colony-stimulating factor receptor family members (*CSF1R*, *CSF2RA*, *CSF3R*), FcγR family members (*FCGR1A* [CD64], *FCGR3A*, *FCGR3B*), killer cell lectin-like receptors (*KLRB1*, *KLRD1*, *KLRK1*), and classic T cell markers (*CD4*, *CD8A*, *CD8B*) in addition to ABC hallmarks (*CD19*^+^, *ITGAX*^+^, and *CR2*^lo^). This broad mRNA immunophenotype included features — *CD19*^+^, *PTPRC* (CD45)^+^, *FCER2* (CD23)^lo^, *CR2* (CD21)^lo^, *ITGA4* (VLA-4)^+^, *ITGAL* (LFA-1)^+^, *TLR4* (CD284)^+^, *CD5*^int^ — consistent with pro-inflammatory GM-CSF^+^ B cells described in MS (103), which are closely related but not identical to innate response activator B cells in mice (104). Given the breadth of the ABC 2 cluster expression profile, we considered whether these cells might be doublets. The numbers of unique features and read counts per cell in ABC 2 were comparable to other phenotypes, consistent with singlets (Supplemental Figure 1B). Consistent with Azimuth scoring, expression of *CD19* and *MS4A1* as well as B lineage transcription factors (e.g., *PAX5*, *BACH2*, *EBF1*) were characteristic of cells in ABC 2. Substantially fewer cells within the cluster expressed non-B lineage transcription factors (e.g., *TCF7*, *GATA3*, *CEBPA*, *GATA2*). Detailed examination of the raw RNA counts for highly expressed cell type biomarkers from eMS ABC 2 cells (*n* = 37 total) identified 28 cells with nonzero *MS4A1* (CD20) versus 2 cells each with nonzero *CD3E* (1 cell also *CD19^+^* and *MS4A1^+^*) and *FCGR1A* (1 cell also *CD19*^+^ and the other *MS4A1*^+^). Finally, reanalysis of the sequencing data with scDblFinder (v1.18; https://github.com/plger/scDblFinder; commit ID 20e4d51) identified only 1 of the 37 eMS cohort barcodes in ABC 2 as a potential doublet. Thus, the ABC 2 cluster had numerous characteristics to support assignment as singlet B cells, though limited presence of other cells may not be excluded definitively.

We next performed GO enrichment of differentially expressed markers between ABCs from eMS→SMSA and eMS→LTNA outcomes (Supplemental Tables 7 and 8). GO terms significantly enriched in eMS→SMSA ABCs were related to B cell activation, signal transduction, immune response, nucleotide biosynthesis, and cellular respiration. By contrast, GO terms enriched in eMS→LTNA ABCs were related to epigenetic modifications, mRNA splicing and transport, and regulation of cellular and mitochondrial autophagy (Supplemental Figure 13A). In addition to being elevated in eMS→LTNA versus eMS→SMSA, numerous genes within these GO categories were depleted in ABCs upon in vitro EBV infection (Supplemental Figure 13B). These included *EZH2*, *BCOR*, and *JMJD6* (related to chromatin modification); *DRB1*, *DDX39A*, and *WBP11* (related to RNA splicing); and *SQSTM1*, *TMEM39A*, and *ULK1* (related to autophagy). Although not associated with any GO term, one of the top transcripts differentially upregulated in eMS→LTNA samples, *MTRNR2L8*, has previously been identified as a biomarker with potential predictive diagnostic value in MS, where reduced expression correlates with disease activity (105) (Supplemental Figure 7A).

**Figure 4:**
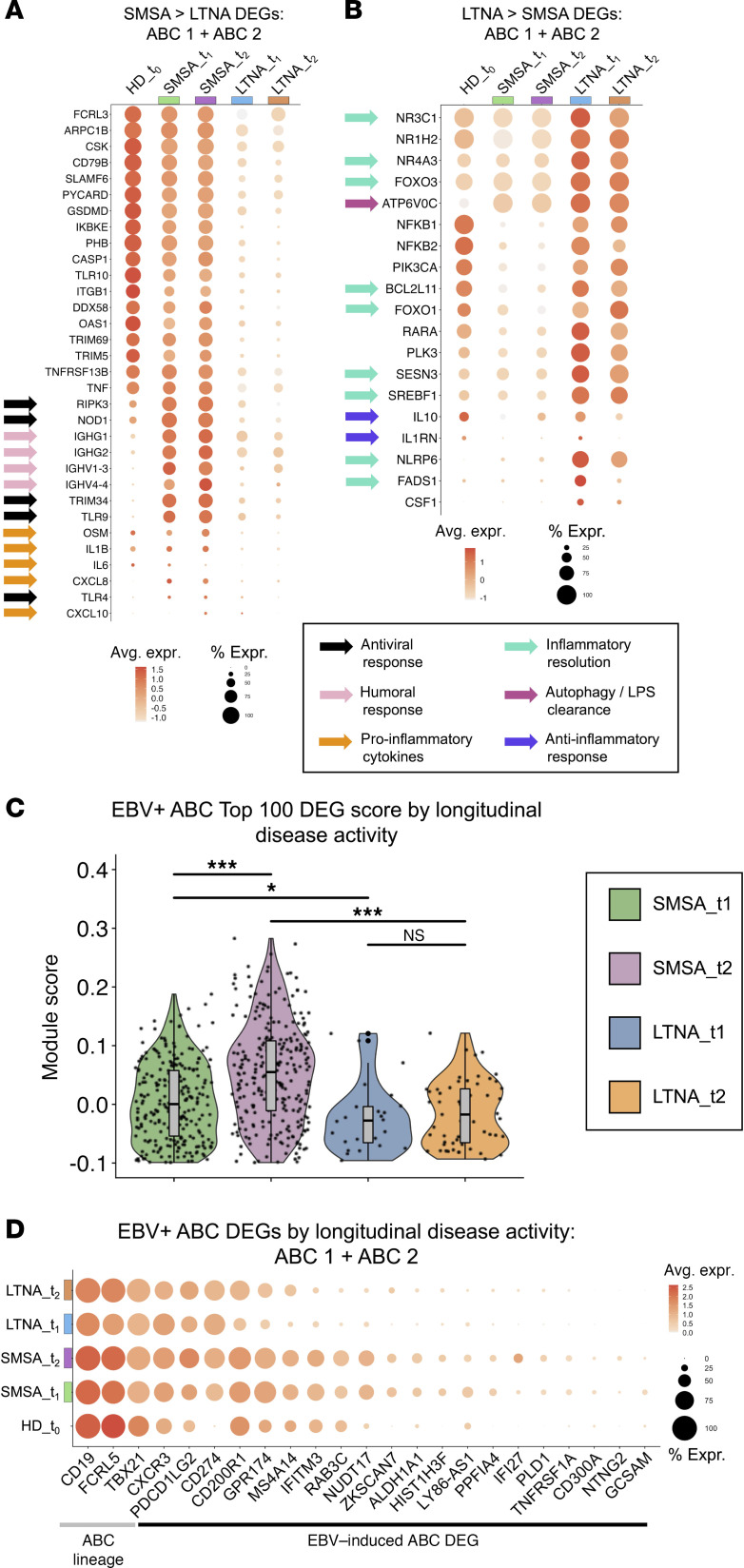
Outcome-stratified functional enrichment of ABCs from people with eMS implicates inflammatory responses linked to antiviral control in MS disease activity. (**A**) Dot plot of GO term–associated genes differentially upregulated in ABCs from eMS→SMSA versus eMS→LTNA. Expression is presented relative to expression in healthy adults (HD_t0). Select genes are denoted by arrows indicating their functional involvement. Dot size represents the percentage of ABCs expressing a gene. Dot color denotes scaled average gene expression. (**B**) Dot plot of GO term–associated genes upregulated in eMS→LTNA versus eMS→SMSA. Expression and gene function annotations are denoted as in **A**. (**C**) Time point– and outcome-stratified scoring of the top 100 EBV^+^ ABC DEGs in patients with eMS. Statistically significant differences in EBV^+^ ABC module score were evaluated by 2-sided Wilcoxon rank-sum test (*P < 0.05; ***P < 0.001). (**D**) Dot plot of select EBV^+^ ABC biomarkers (DEGs) stratified by time point and outcome in ABCs from people with eMS. Several ABC lineage genes are shown in addition to EBV-associated DEGs. Cell frequency and expression level are presented as in **A** and **B**.

Further examination of differential expression between SMSA and LTNA outcomes in ABC 1 and ABC 2 subsets highlighted differential pathogenic potential. ABCs from eMS→SMSA exhibited higher expression of genes involved in antiviral sensing (*RIPK3*, *NOD1*, *TRIM34*, *TLR9*, *TLR4*) and pro-inflammatory (*OSM*, *IL1B*, *CXCL8*) responses (Figure 4A and Supplemental Figure 14). Elevated *NOD1*, *TLR9*, and *TLR4* expression in these cells supports an enhanced capacity for inflammatory responses to pathogen-associated molecular patterns. Similarly, *RIPK3* and *IL1B* expression in eMS→SMSA ABCs was noteworthy given the role of RIPK3 in NLRP3 inflammasome activation and subsequent IL-1B activation (106, 107). By contrast, ABCs from eMS→LTNA samples were defined by elevated expression of genes with roles in limiting inflammatory responses (Figure 4B and Supplemental Figure 14). These included *NR4A3*, which antagonizes NF-κB signaling (108, 109). *FOXO3* is essential for the induction of autophagy (110), which limits inflammatory responses by targeting activated inflammasomes (111). Moreover, *NLRP6* expression was consistent with negative regulation of inflammation in the context of innate immune responses (112). GO analysis of genes differentially elevated in ABC 2 versus ABC 1 in people with eMS identified terms related to immune cell activation, cytotoxic degranulation, IL-1β and TNF cytokine production, and TLR signaling pathways (Supplemental Figure 15). Thus, expression in ABC subsets from eMS→SMSA versus eMS→LTNA, especially ABC 2 cells, support differential outcomes related to control of cellular inflammatory responses, possibly resulting from pathogenic stimulation. Despite the lack of detected EBV reads, stratifying ABCs from people with eMS according to clinical outcome (SMSA versus LTNA) revealed significantly higher expression of the EBV^+^ ABC DEG module in samples from individuals exhibiting subsequent disease activity. Moreover, EBV^+^ ABC module scores were significantly higher in t_2_ versus t_1_ sample ABCs from people with eMS who experienced SMSA but not in LTNA (Figure 4C). Numerous EBV-induced ABC DEGs including *NUDT17*, *ALDH1A1*, *IFI27*, and *TNFRSF1A* were enriched in eMS→SMSA versus eMS→LTNA (Figure 4D).

While the ABC 2 cellular phenotype was rare, it was nearly 7-fold more frequent in eMS→SMSA than eMS→LTNA samples after accounting for sample sizes (~1 in 330 B cells in eMS→SMSA, ~1 in 2,500 B cells in eMS→LTNA). Overall, 36 cells from 8 of 13 people with eMS→SMSA activity exhibited the ABC 2 phenotype, compared with only 1 cell from 1 of 3 eMS→LTNA cases. Based on the number of ABC 2 cells expected from eMS→LTNA by scRNA-Seq library sizes, the probability of observing ≤1 cell by random chance alone was less than 1 in 50 (0.0082 < *P*_Poisson_ < 0.0198; Supplemental Figure 16). This differential ABC 2 frequency between eMS→SMSA and eMS→LTNA further indicates ABC 2 contains singlets, since library prep and pooled sequencing of all eMS samples would minimize sample-dependent (outcome-dependent) doublet rates. Thus, the inflammation-associated ABC 2 phenotype was significantly underrepresented in LTNA versus people with subsequent MS activity.

We next used informatic methods to explore potential viral contribution to the observed eMS and EBV^+^ ABC phenotype. We used *cis*-regulatory prediction (113) to identify genes associated with EBNA binding sites detected using ChIP-Seq and cross-referenced these predictions against genes upregulated in eMS and EBV^+^ ABCs relative to resting cells (Supplemental Figure 17A). This yielded 82 EBNA-associated genes, including *TBX21*, *CXCR3*, *FCRL2*, *FCRL5*, *FCGR2A*, *AREG*, *CCL5*, *CD69*, *CD84*, *CD300A*, *FGR*, *GPR174*, *HCK*, and *TBKBP1*. These 82 genes constituted modest enrichment of GO terms including defense response (GO:0006952, *n* = 17 genes, FDR = 0.0442), leukocyte activation (GO:0045321, *n* = 14 genes, FDR = 0.0442), regulation of cell migration (GO:0030334, *n* = 13 genes, FDR = 0.0468), and leukocyte degranulation (GO:0043299, *n* = 10 genes, FDR = 0.0468). Analysis of the *TBX21* locus in the LCL GM12878 revealed accessible chromatin with activating histone marks (H3K9ac, H3K4me3, H3K27ac), RNA polymerase II, and binding sites for EBNA-LP and EBNA2 at the *TBX21* transcription start site (Supplemental Figure 17B). Collectively, informatic insights suggested that EBV-encoded transcriptional cotransactivators may mediate *TBX21*/T-bet expression and thereby affect ABC functions and responses. We also examined expression of cellular genes previously identified as biomarkers of EBV lytic reactivation (114) (Supplemental Figure 18). With few exceptions, biomarkers of the EBV lytic phase were strongly and broadly expressed in ABC 2. Collectively, these data suggest indirect roles of EBV infection in potentiating ABC pathogenicity. Without excluding other possible mechanisms, virus-related ABC responses may contribute to eMS and MS disease activity via inflammatory cytokine production.

### Validation of ABC signature by flow cytometry of expanded eMS patient cohort.

**Figure 5:**
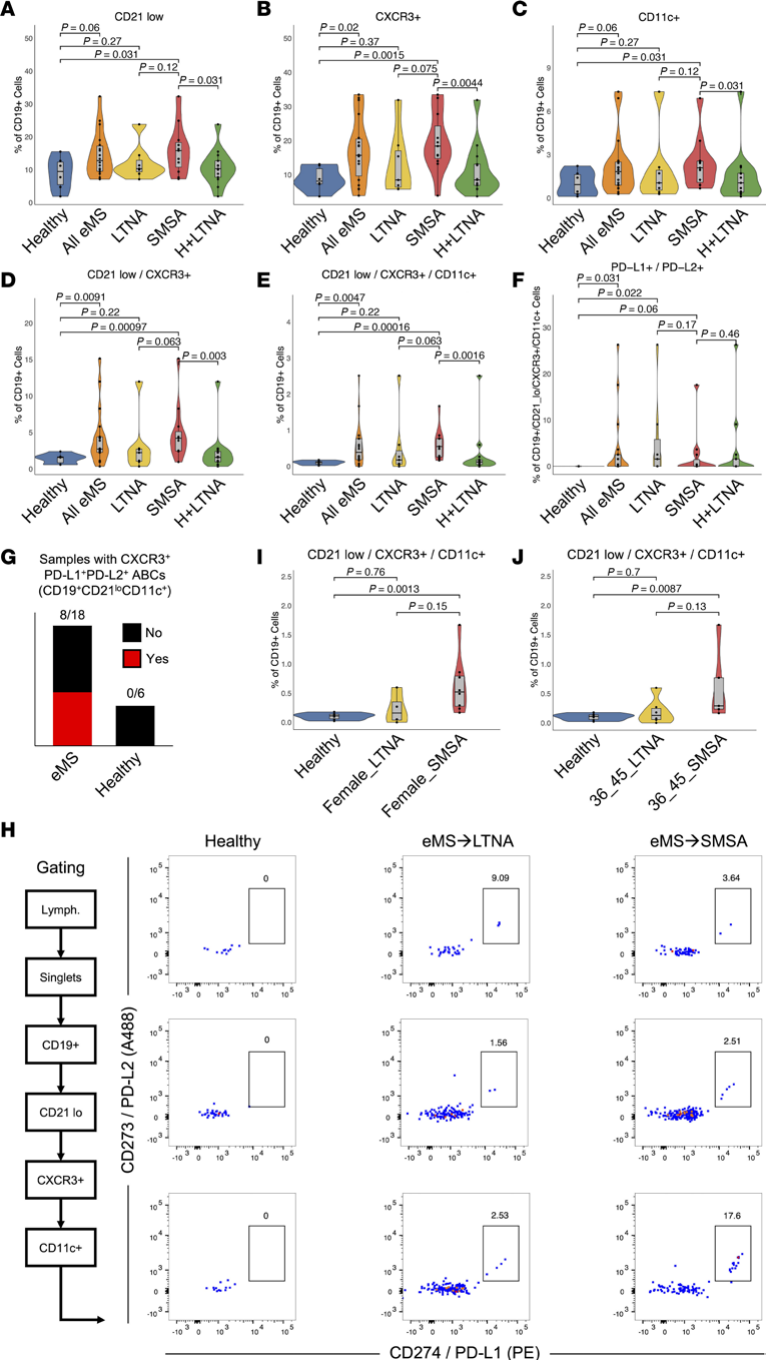
Flow cytometry validation of peripheral ABC expansion and immune checkpoint expression in people with eMS. (**A**) Quantification and statistical comparison of CD19^+^CD21^lo^ cells among healthy (*n* = 6), all eMS (*n* = 18), eMS→LTNA (*n* = 7), eMS→SMSA (*n* = 11), and healthy + LTNA (*n* = 13) groups. All statistics represent *P* values from 1-sided Wilcoxon rank-sum tests. (**B**) Quantification and statistical comparison of CD19^+^CXCR3^+^ cells between groups as shown in **A**. (**C**) Quantification and statistical comparison of CD19^+^CD11c^+^ cells between groups as shown in **A** and **B**. (**D**) Quantification and statistical comparison of CD19^+^CD21^lo^CXCR3^+^ cells between groups as shown in **A**–**C**. (**E**) Quantification and statistical comparison of ABCs (CD19^+^CD21^lo^CXCR3^+^CD11c^+^) cells between groups as shown in **A**–**D**. (**F**) Quantification and statistical comparison of PD-L1^+^PD-L2^+^ ABCs (CD19^+^CD21^lo^CXCR3^+^CD11c^+^) between groups as shown in **A**–**E**. (**G**) Summary of eMS and healthy samples exhibiting any PD-L1^+^PD-L2^+^ expression in CXCR3^+^ ABCs (CD19^+^CD21^lo^CD11c^+^). The median frequency of such cells in positive cases is approximately 1 per 10,000 total B cells. (**H**) PD-L1 (CD274) and PD-L2 (CD273) coexpression in CD19^+^CD21^lo^CXCR3^+^CD11c^+^ cells from select eMS→LTNA and eMS→SMSA but not healthy donors. (**I**) Sex-matched (female-only) quantification and statistical comparisons of CD19^+^CD21^lo^CXCR3^+^CD11c^+^ cells among healthy (*n* = 6), eMS→LTNA (*n* = 4), and eMS→SMSA (*n* = 8) groups. Statistics represent *P* values from 2-sided Wilcoxon rank-sum tests. (**J**) Age-matched (36- to 45-year-olds) quantification and statistical comparisons of CD19^+^CD21^lo^CXCR3^+^CD11c^+^ cells among healthy (*n* = 6), eMS→LTNA (*n* = 6), and eMS→SMSA (*n* = 5) groups. Statistics represent *P* values from 2-sided Wilcoxon rank-sum tests.

An expanded surface biomarker panel was applied to t_2_ PBMC samples from 18 participants in the original STAyCIS Trial (*n* = 7 LTNA, *n* = 11 SMSA outcomes) and healthy donors (*n* = 6). In addition to ABC markers and CXCR3, we evaluated PD-L1 (CD274) and PD-L2 (CD273) expression, since these genes were induced by EBV at the mRNA level and expressed in eMS ABCs from scRNA-Seq (Figure 5 and Supplemental Figure 19). The CD21^lo^ fraction of the B cell compartment was significantly expanded in people with eMS who experienced subsequent MS activity versus healthy controls (1-sided Wilcoxon *P* = 0.031). CXCR3^+^ and CD11c^+^ B cell subsets were likewise enriched in eMS→SMSA outcomes. Similar results were observed for comparisons of SMSA to grouped healthy and LTNA samples (Figure 5, A–C). Moreover, eMS cohort samples contained significantly more CD21^lo^CXCR3^+^ B cells (4.2% ± 3.9% versus 1.4% ± 0.7%, Wilcoxon *P* = 0.0091) and CD21^lo^CXCR3^+^CD11c^+^ B cells (0.58% ± 0.63% versus 0.10% ± 0.06%, Wilcoxon *P* = 0.0047) than healthy controls. CD21^lo^CXCR3^+^ B cells and CD21^lo^CXCR3^+^CD11c^+^ B cells were likewise significantly enriched in eMS→SMSA outcomes versus healthy controls and all non-MS outcomes (healthy controls plus eMS→LTNA outcomes, *n* = 13), though differences between eMS→LTNA and eMS→SMSA outcomes were not significant (Figure 5, D and E). Thus, MS activity following initial presentation was associated not only with ABC enrichment specifically but also with broader enrichment of CXCR3^+^ and CD21^lo^ cells across peripheral B cell niches. We also found that CXCR3^+^ ABCs (CD19^+^CD21^lo^CD11c^+^) coexpressing the immune checkpoint ligands PD-L1 and PD-L2 were significantly enriched in people with eMS versus healthy controls (Wilcoxon *P* = 0.031; Figure 5F). Specifically, CD19^+^CD21^lo^CXCR3^+^CD11c^+^ cells from 8 of 18 eMS samples contained a PD-L1^+^PD-L2^+^ subset compared with 0 of 6 healthy controls (Figure 5, G and H). Comparison of sex-matched samples identified enrichment of CD21^lo^CXCR3^+^CD11c^+^ B cells from eMS→SMSA (*n* = 8) versus healthy controls (*n* = 6; Wilcoxon test *P* = 0.0013) and eMS→LTNA (*n* = 4; Wilcoxon *P* = 0.15) in female donors (Figure 5I). Similar ABC enrichment was observed in age-matched cohorts (36 to 45 years; eMS→SMSA *n* = 5; eMS→LTNA *n* = 6) (Figure 5J). Case-matched samples (*n* = 4 individuals) from eMS baseline and 6-month follow-up longitudinally exhibited elevated CD11c^+^, CXCR3^+^, and PD-L1^+^PD-L2^+^ subsets of CD21^lo^ B cells, though these differences were not statistically significant (Supplemental Figure 19, B and C).

Collectively, flow cytometry validation experiments verified that ABCs are peripherally expanded in eMS — particularly in cases of additional MS activity — relative to healthy donors. These experiments further supported ABC upregulation of certain lineage-ectopic genes and critical immune checkpoint molecules associated with responses to EBV in a subset of eMS cases. Elevated PD-L1 in eMS versus healthy ABCs is particularly noteworthy given the recent identification of EBV^+^ PD-L1 cells in postmortem chronic active MS lesion tissue (115). However, we cannot definitively determine whether ABC responses in eMS are strictly dependent on direct EBV infection or reflect bystander effects based on our data.

## Discussion

In summary, this study provides single-cell transcriptomic and protein-level evidence of ABCs with pathogenic features in people with eMS. These cells, which constituted about 5% of peripheral B cells, share many characteristics with ABCs in other disease contexts. Notably, eMS ABCs were distinguished from ABCs in other diseases by transcriptomic similarities to ABCs in response to EBV infection, which were identified in a prior in vitro study (34). Although EBV reads were not directly detected in eMS ABCs, we further found that the EBV-associated ABC gene signature was enriched in people with eMS who experienced additional disease activity versus individuals who did not during the STAyCIS Trial. A smaller subset of *CD19^+^CD20^+^TBX21^+^ITGAX^+^* cells (ABC 2) from people with eMS (~0.3% of B cells) that exhibited surprisingly broad immune lineage expression and pro-inflammatory cytokine transcripts was also underrepresented in people without subsequent disease activity after baseline.

Prior studies have separately demonstrated EBV-induced CXCR3 expression in B cells (48), positive correlation between CXCR3^+^ B cell frequencies and EBV viral load in people with MS (64), and T-bet^+^CXCR3^+^ B cell induction and neuroinvasion in people with MS (50). In conjunction with these findings, the present identification of a shared gene signature — including CXCR3 — in EBV^+^ ABCs and ABCs from people with eMS absent in healthy controls further implies a link between neuroinvasive CXCR3^+^ ABCs and EBV infection. However, it is unclear whether such association may be direct or indirect, and definitive causality cannot be established in the absence of consistent viral detection. The enrichment of neuronal genes including cell adhesion molecules in the EBV-associated ABC signature is consistent with an acquired neurotropic capacity that may facilitate CNS infiltration as well as localized viral antigen presentation and inflammatory responses that precipitate MS. Because ABCs differentiate into PBs in response to innate and inflammatory stimuli (9), it is further conceivable that EBV infection could promote low-affinity antibody production by activated ABCs in the CNS. The likelihood of this pathogenic sequence of events would partially depend on the frequency of ABCs, which increases with age and is elevated in genetic females (1). In summary, this model is consistent with epidemiological aspects of MS, including age at time of EBV seroconversion and age- and sex-dependent accumulation of ABCs. Likewise, the efficacy of anti-CD20 therapy (116) and clinical exacerbations observed upon IFN-γ treatment (117) are each consistent with pathogenicity of CD20^+^ ABCs, which proliferate and differentiate in response to IFN-γ. Notably, this model also aligns with empirical findings and prevailing hypotheses (55, 118, 119) of EBV etiology in MS: molecular mimicry (56), pro-inflammatory bystander damage (94, 103), and the potential for antibody production by autoreactive clones, the latter of which is a frequent fate of extrafollicular activated ABCs (12, 13).

Detection of EBV genomes, transcripts, and proteins has been reported in MS brain lesions; however, negative results in multiple studies — in some cases using the same samples — precludes definitive functional insights linking viral antigens to MS pathology (58–63). The same is true regarding the lack of detected EBV reads within previous findings from an scRNA-Seq study of people with MS (49). Similarly, the present study did not directly detect EBV in B cells from early MS clinical samples. This may be due to one or more factors, including transcriptome undersampling inherent to current scRNA-Seq methods (120); inefficient capture chemistry for the most abundant EBV transcripts, which are not polyadenylated (121); limited viral gene expression owing to restricted latency programs or failure to establish successful infection (34, 122–124); or a combination thereof. In this case, the transcriptomic profile exhibited by ABCs in eMS and in response to EBV infection in vitro may reflect epigenetically encoded responses of the ABC niche that EBV, a prevalent stimulus, is capable of eliciting. Likewise, although our prior work demonstrated that EBV can infect ABCs based on viral read detection (34), we cannot definitively rule out the possibility that EBV-induced responses of other infected cells (e.g., inflammatory cytokine production) might indirectly activate bystander ABCs that do not harbor the virus.

There are several limitations to this study. Primarily, our findings do not definitively identify EBV mechanistic involvement in early MS. Specifically, there is a current lack of insight with respect to (a) whether the observed age dependence of EBV seroconversion for risk in MS is directly related to the increase in ABC frequency over time; (b) whether EBV promotes or otherwise enhances the accumulation of ABCs in vivo; and (c) whether periodic EBV reactivation can result in chronic de novo infection of ABCs or other B cell subtypes, consistent with autoimmune disease flares. The number of LTNA samples analyzed with scRNA-Seq was small; however, flow cytometric validation of the ABC enrichment and checkpoint molecule expression in a larger number of LTNA cases provides additional support for the observed results. Also, this study did not examine patient- and time point–matched CSF samples because of limited availability. Future efforts to contextualize ABC expansion and EBV biomarkers in peripheral blood with biomarker signatures in CSF will be highly valuable. Additional studies are required to investigate the consequences of EBV infection in different genetic backgrounds with known autoimmune susceptibility variants. Thus, the nature of EBV infection in promoting the formation and activation of ABCs and the relevance of this host-virus relationship in MS and other autoimmune diseases warrants substantial focus in future work. As noted previously, samples analyzed herein were obtained from individuals diagnosed with CIS using the 2001 McDonald criteria, many of whom would likely have been diagnosed with MS at baseline using current standards. Thus, future studies should examine the associations and pathologic involvement of ABCs on a larger scale in clinical cohorts of people with eMS using updated diagnostic criteria.

Despite these unknowns, the present work advances our understanding of alterations to the peripheral B cell landscape in patients during the early stages of MS, biomarkers of peripheral B cells to distinguish differential disease activity in MS, and evidence of pro-inflammatory ABC expansion and immune checkpoint evasion. Likewise, the data herein underscore the value and importance of addressing questions of viral pathogenesis related to disease at cellular resolution with high-dimensional techniques.

## Methods

### Sex as a biological variable.

Our gene expression study included 32 human PBMC samples from 16 individuals. As expected given the distribution of the disease under study, these were split as 11 females and 5 males. More demographic details regarding the trial and this specific cohort are noted below.

### Statistics.

Statistical comparisons within this study were made as described above and as denoted in the text. Broadly, genes found to be differentially expressed in this study were identified based on significance after multiple-hypothesis testing (Bonferroni’s correction) using the Seurat FindMarkers function. Further, statistical significance of differential expression levels for select genes of interest expression were calculated via 2-sided Wilcoxon rank-sum test. Significant differences in gene expression distributions were determined via 2-sided KS test. Significant differences in cell phenotype frequencies (measured by scRNA-Seq, CITE-Seq, and flow cytometry) between groups were determined using Wilcoxon rank-sum tests. *P* < 0.05 was considered statistically significant.

### Study approval.

The original STAyCIS Trial received institutional review board (IRB) approval from 14 centers in the United States and Canada. Single-cell analyses of samples from the STAyCIS Trial were additionally approved by a Duke University IRB. All participants provided written informed consent prior to study entry.

### Data availability.

All clinical data associated with the STAyCIS Trial are publicly available on the TrialShare website, https://www.itntrialshare.org/project/Studies/ITN020AIPUBLIC/Study%20Data/begin.view B cell scRNA-Seq + CITE-Seq data from the eMS cohort reported herein are deposited in the National Center for Biotechnology Information Gene Expression Omnibus (accession GSE267750). Additional methods are available in Supplemental Methods.

## Author contributions

EDS, SGG, and MAL conceived and designed the study and wrote the manuscript. E Haukenfrers, VJ, JG, KA, and E Hocke performed single-cell experiments and curated public datasets. EDS and VJ processed the data and extracted B cell populations. EDS analyzed the sequencing data. EDS and GQH performed flow cytometry and RNA Flow-FISH experiments and analyses. LAC, KMH, and SSZ reviewed and edited the manuscript. All authors have approved the final version of the manuscript.

## Supplementary Material

Supplemental data

Supplemental tables 1-8

Supporting data values

## Version 1 05/13/2025

In-Press Preview

## Version 2 06/23/2025

Electronic publication
