# Supplementary material for: Early multiple sclerosis activity associated with TBX21+CD21loCXCR3+ B cell expansion resembling EBV-induced phenotypes
Source: JCI Insight. 2025 May 13;10(12):e188543. doi: 10.1172/jci.insight.188543 (PMC12220969; doi:10.1172/jci.insight.188543)
Supplement: Supplemental data [file jciinsight-10-188543-s040.pdf]

SUPPLEMENTARY METHODS

Clinical study design (ITN STAyCIS Trial)

CIS PBMC samples used in this work were originally collected as part of the Immune Tolerance Network (ITN) STAyCIS Trial (1). The clinical objective of the original STAyCIS Trial was to evaluate the safety and efficacy of Atorvastatin in CIS. A total of 81 people with high risk of conversion to MS were enrolled upon initial CIS diagnosis (per contemporaneous diagnostic guidelines) in a two-arm clinical trial containing experimental (Atorvastatin, n=49) and control (placebo, n=32) treatment groups. The average age of the full cohort was 34 years, and the average time since CIS onset to screening was 65 days. All participants had no previous history of neurological disease and were seen within 210 days of the neurologic event. PBMC samples were acquired at two timepoints following initial CIS diagnosis: t_1 (baseline visit; at least 28 days after completion of corticosteroid treatment and within 210 days of CIS presentation) and t_2 (at least 3 months post-t_1; mean=172 days post-t_1, range=84-357 days). Blood samples were collected and purified to PBMCs from each patient at each endpoint. We note that the criteria for MS diagnosis have been relaxed since the time of the STAyCIS study. Thus, by current standards some of the people in the CIS→MS group (but not CIS→LTNA) would have been diagnosed with MS at baseline. We have chosen to refer to the original diagnoses of CIS because this terminology accurately reflects the application of the criteria (2) that were available and up to date at the time of the STAyCIS trial. As a note, “definite MS” was one of the exclusion criteria for enrollment in the STAyCIS study.

Patient samples for scRNA / CITE-seq

PBMCs from sixteen people at each of two timepoints (n=32) were selected for single-cell sequencing. Eleven of the selected individuals were female (age=32 □ 8.2 years) and five were male (age=38 □ 6.4 years). Of these people, eight met the defined primary endpoint for MS diagnosis within the 12 month treatment period and two participants met the endpoint criteria within 18 months from t_1. Three additional participants subsequently met these criteria after the 18 month study period. The remaining three participants from the cohort analyzed herein were characterized as long-term no evidence of activity (LTNA) based on the absence of new T2 lesion, Gd-enhancing lesions, and no clinical exacerbations (3/16 selected cases).

Single-cell library preparation

PBMCs were incubated with a panel of 18 antibodies with unique conjugated sequencing tags (antibody-derived tags, ADTs) for surface protein expression profiling. The panel contained

antibodies against the following surface markers: CD3, CD4, CD8, CD11b (ITGAM), CD11c (ITGAX), CD14, CD16 (FCGR3A), CD19, CD25 (IL2RA), CD27, CD45 (PTPRC), CD56 (NCAM1), CD64 (FCGR1A), CD196 (CCR6), CD294 (PTGDR2), CD366 (HAVCR2), KLRG1, and TCRVa24-JA18. Following ADT labeling, cells were prepared as single-cell RNA-seq (scRNA-seq) libraries using the 10x Genomics Chromium Controller and Single-Cell Gene Expression kit with v3.1 chemistry (5' v1 single index). Cells were normalized to $1x10^3$ cells/□l, viability assessed, and titered to maximum of ~10,000 cells per library. Cells were resuspended in master mix containing reverse transcription reagents and combined with gel beads carrying the sequencing primers, barcodes, unique molecular identifier, and a poly-dT primer for RT. Full length cDNAs were cleaned and assayed to ensure lengths between 200-5000bp. Enzymatic fragmentation of the cDNA was used prior to adapter and sample index ligation; TruSeq read 2 primers were added via End Repair, A-tailing, Adaptor Ligation, and PCR. qPCR will be used to assess P5 and P7 adapter ligation, prior to size assessment of between 400-500bp. We generated 150bp paired end sequencing reads on an Illumina NovaSeq6000 (read 1=26 bp, read 2=91 bp, index read=8 bp) at a target of 50,000 reads/cell for gene expression and 5,000 reads/cell for ADT libraries. Output base calls (.bcl format) from sequencing were used to assemble single-cell RNA and ADT reads.

Read assembly, QC, and alignment

We first demultiplexed raw FASTQ reads and aligned them to the human transcriptome before using Seurat (3) to perform QC and analyze data by normalizing on a log scale after filtering for minimum gene and cell frequency cut-offs (4). We identified and exclude possible multiplets (4) and reduced noise by removing technical artifacts using regression methods (4). Principal components (PCs) were calculated using the most variably expressed genes in our dataset (4) and examined for cell dividing states (5). Significant PCs were determined (5) and carried forward for cell clustering and to enhance visualization (4).

Data processing and visualization

Upon completing the steps described above, we obtained ~9,000 cells on average (8,949 □1,559) from each of the 32 longitudinal CIS samples. Count matrices were prepared as a Seurat (6, 7) object containing 274,277 PBMCs after QC filtering to exclude cells with fewer than 200 unique feature RNAs. Data were log normalized and scaled prior to identification of the top 2,000 variable features as per standard Seurat workflows. Normalized and scaled data were dimensionally reduced through principal component analysis (*RunPCA*). Cell neighborhood

identification, UMAP projection, and unbiased clustering were performed (*FindNeighbors*, *RunUMAP*, *FindClusters*). Automated cell type annotation with Azimuth (8) delineated 14,297 B cells (Naïve B, Intermediate B, Memory B, and Plasmablasts), which were utilized in subsequent analyses. Adaptive low-rank approximation (ALRA) was used at this stage to generate an imputed gene expression matrix with preserved biological zeros (true absence of transcript expression) and correction of technical transcript dropout (9). Imputed expression values are presented unless otherwise stated. Cell cycle scoring and phase were also assigned based on expression of curated S- and G_2/M-phase gene sets.

Additional scRNA-seq dataset provenance and curation

In addition to the newly generated CIS cohort data described above, we collected the following publicly available scRNA-seq datasets for autoimmune diseases and chronic infections: GSE133028 and GSE138266 (MS and healthy controls, PBMC) (10, 11); GSE157278 (PSS, PBMC) (12); GSE135779 (SLE and healthy controls, PBMC) (13); GSE149729 (Malaria, HIV, and healthy controls, peripheral B cells) (14); and GSE189141 (*in vitro* EBV infection, peripheral B cells) (15). For the *in vitro* early infection study (GSE189141), primary B cells from two independent donors were collected before (day 0) and after (days 2, 5, and 8) infection with the B95-8 strain of EBV at MOI=5.

Single cell count matrices from each of these studies were prepared as Seurat objects, from which B cell subsets were identified and extracted as described above for the CIS cohort data. After curation, B cells from all datasets were integrated with batch correction into a single object using integration feature selection and anchor identification with Seurat.

Analysis, statistical methods, and simulation

Differentially expressed genes between cell phenotypes, disease, annotated clusters, and longitudinal outcome were identified using the *FindMarkers()* function in Seurat. For most comparisons, differentially expressed genes were returned if expressed in a minimum of 50% of cells in the group of interest with an average $\log_2$ fold change > 0.6. Differential expression data are included as supplementary data files, including calculated p-values with and without multiple hypothesis (Bonferroni) correction. We observed that some genes were identified as differentially expressed in select comparisons across diseases due to discrepancies in gene symbol annotation from publicly available datasets. This resulted from alignment of available SLE data against hg19, whereas other datasets were aligned against hg38 (or hg38 + type 1 EBV, in the case of CIS and EBV *in vitro* reads). Such genes were excluded from analysis and figures by only considering

transcripts with conserved annotation across all integrated datasets. We note that this is a conservative approach that ensures the fidelity of presented data but may fail to identify additional differentially expressed genes across ABCs from SLE versus other diseases. Critically, differential reference alignment had no effect on comparisons made within CIS cohort B cells or between CIS and EBV^+ B cells. For certain analyses, expression of curated genes of interest previously identified from ABCs before and after EBV infection *in vitro* (using the same approach) were analyzed. As an example, the top 100 DEG lists for various EBV^+ B cell phenotypes were derived from significant DEGs (by Wilcox test with Bonferroni correction) identified from Seurat *FindMarkers()* tests of one-versus-all expression in clusters from early EBV infection data. DEGs between EBV^- ABCs and EBV^+ ABCs were similarly calculated from the early infection dataset. For more detail on phenotypes of EBV^+ and EBV^- B cell niches, readers are referred to the original time-resolved study of early infection (15).

For select genes of interest, statistical significance of differential gene expression across groups was calculated via two-sided Wilcoxon rank-sum test (and, in some cases, further validated via two-sided Kolmogorov-Smirnov (KS) test). Poisson simulation was used to randomly sample cells from empirically observed cell frequencies per individual to evaluate outcome representation probabilities within specific phenotypes of interest. Cumulative Poisson probabilities were calculated for empirically observed cell frequency by phenotype in addition to expected frequencies (Poisson □ parameter).

Gene ontology enrichment

Gene ontology (GO) biological process (BP) enrichment analysis was performed from differentially expressed gene sets using the *enrichGO* function from clusterProfiler (16). Genes with $\log_2$ fold change values > 0.6 in a given comparison were considered for this analysis, using the whole genome as a background. Resulting terms were filtered to include those with Bonferroni-adjusted $p < 0.05$ and $q < 0.1$. Significantly enriched GO BP terms are presented as bar (barplot), dot (dotplot), and network (emmapplot) plots to represent significance and the number of genes per term set.

Antibodies

The following antibodies were used in flow cytometry or RNA-FiSH experiments: anti-CD19 BV421 (clone HIB19, cat. #302234, BioLegend, San Diego, CA); anti-CXCR3 APC (clone G025H7, cat. #353708, BioLegend); anti-CD21 PE-Cy7 (clone Bu32, cat. #354912, BioLegend); anti-CD274 (PD-L1) PE (clone 29E.2A3, cat. #329705, BioLegend); anti-CD273 (PD-L2) Kiravia

Blue 520 (clone 24F.10C12, cat. #329629, BioLegend); and anti-CD11c APC Fire 750 (clone S-HCL-3, cat. #371509, BioLegend).

Flow cytometry

Additional cryopreserved PBMC samples from eighteen people in the CIS cohort (n=11 MS; n=7 LTNA) were used for flow cytometric validation. Post-baseline samples were analyzed for all cases (6-month follow-up, except one 3-month follow-up for which a 6-month sample was not available), and baseline samples were analyzed where available. Peripheral B cells (in whole PBMCs or negatively selected CD19$^+$ fractions) from healthy controls (n=6) were also evaluated. Briefly, cells were thawed in a 37°C water bath, resuspended to 10 mL in RPMI + 20% heat-inactivated (HI) FBS (R20), and evaluated for viability by Trypan Blue staining. Cells were washed twice by centrifugation at room temperature (5 min @ 350 x g) and resuspension in flow cytometry buffer (1x PBS + 2% HI FBS). Washed cell pellets were resuspended with fluorescent antibody master mix (CD19-BV421, CXCR3-APC, CD21-PE_Cy7, CD274-PE, CD273-KIRA_Blue_520, CD11c-APC_Fire750) and incubated in the dark for 30 min at room temperature. Unstained cells, n minus 1 (n-1) antibody stained controls, and single-stain compensation beads were also prepared and analyzed. After staining, samples were washed twice by centrifugation and resuspension in flow cytometry buffer. Resuspended cells were analyzed using a Canto II cytometer (BD Biosciences, Franklin Lakes, NJ). Exported .fcs files were gated and analyzed with FlowJo (v10, BD Biosciences), and cell population frequencies were statistically evaluated.

Cytokine assays

27-plex Luminex cytokine/chemokine secretion profiling was performed from 48h supernatants of resting peripheral blood CD19$^+$ B cells, early EBV$^+$ B cells that had undergone at least one population doubling after infection (□PD1), and EBV-immortalized LCLs (as in Price *et al.* (17)) . Two technical replicates from each of two biological donors were prepared and analyzed for these experiments. Resting B cells were purified from PBMCs by negative isolation (BD iMag kit, BD Biosciences). Early EBV$^+$ cells (□PD1) were sorted by FACS at 6 days post-EBV infection of PBMCs via CD19 positivity and CFSE staining to track cell proliferation status. Sorted EBV$^+$ cells were then re-cultured for two days, after which supernatants were harvested for analysis. LCLs were grown out from PBMC infections with limiting virus dilution and analyzed at five weeks post-transformation. Each of the three sample types were pure B cell populations. The cytokine panel detected the following secreted proteins: PDGFB, IL-1□, IL-1RA, IL-2, IL-4, IL-5, IL-6, IL-7, IL-8, IL-9, IL-10, IL-12, IL-13, IL-15, IL-17, CCL11 (Eotaxin), FGF2, G-CSF, GM-CSF, IFN□,

CXCL10 (IP-10), CCL2 (MCP-1), CCL3 (MIP-1□), CCL4 (MIP-1□), CCL5 (RANTES), TNF□, and VEGF. Statistically significant differences in cytokine secretion between resting B cells and □PD1 or LCLs were determined via two-sided Wilcoxon rank-sum tests.

ChIP-seq gene regulatory prediction

The Gene Regulatory Enrichment Analysis Tool (GREAT) (18) was used to predict *cis*-linked genes to binding sites for EBNA-2, EBNA-3A, and EBNA-LP identified from ChIP-seq experiments in the GM12878 LCL (19, 20). Linked gene predictions were cross-referenced against genes that exhibited significant upregulated expression in both CIS and EBV^+ ABCs relative to resting ABCs. Gene network analysis for EBNA-associated differentially expressed genes was performed using Cytoscape (21), and EBNA as well as other epigenetic ChIP signals and bulk chromatin accessibility (22) were visualized using IGV (23).

Supplementary References

1. Waubant E, Pelletier D, Mass M, Cohen J, Kita M, Cross A, et al. Randomized controlled trial of atorvastatin in clinically isolated syndrome: the STAyCIS study. *Neurology.* 2012;78(15):1171-8.
2. McDonald WI, Compston A, Edan G, Goodkin D, Hartung HP, Lublin FD, et al. Recommended diagnostic criteria for multiple sclerosis: guidelines from the International Panel on the diagnosis of multiple sclerosis. *Annals of Neurology: Official Journal of the American Neurological Association and the Child Neurology Society.* 2001;50(1):121-7.
3. Satija R, Farrell JA, Gennert D, Schier AF, and Regev A. Spatial reconstruction of single-cell gene expression data. *Nat Biotechnol.* 2015;33(5):495-502.
4. Satija R. Seurat - Guided Clustering Tutorial. http://satijalab.org/seurat/pbmc-tutorial.html.
5. Macosko EZ, Basu A, Satija R, Nemesh J, Shekhar K, Goldman M, et al. Highly Parallel Genome-wide Expression Profiling of Individual Cells Using Nanoliter Droplets. *Cell.* 2015;161(5):1202-14.
6. Stuart T, Butler A, Hoffman P, Hafemeister C, Papalexi E, Mauck III WM, et al. Comprehensive integration of single-cell data. *Cell.* 2019;177(7):1888-902. e21.
7. Satija R, Farrell JA, Gennert D, Schier AF, and Regev A. Spatial reconstruction of single-cell gene expression data. *Nature biotechnology.* 2015;33(5):495-502.
8. Hao Y, Hao S, Andersen-Nissen E, Mauck III WM, Zheng S, Butler A, et al. Integrated analysis of multimodal single-cell data. *Cell.* 2021.
9. Linderman GC, Zhao J, Roulis M, Bielecki P, Flavell RA, Nadler B, et al. Zero-preserving imputation of single-cell RNA-seq data. *Nature Communications.* 2022;13(1):1-11.
10. Ramesh A, Schubert RD, Greenfield AL, Dandekar R, Loudermilk R, Sabatino JJ, et al. A pathogenic and clonally expanded B cell transcriptome in active multiple sclerosis. *Proceedings of the National Academy of Sciences.* 2020;117(37):22932-43.
11. Schafflick D, Xu CA, Hartlehnert M, Cole M, Schulte-Mecklenbeck A, Lautwein T, et al. Integrated single cell analysis of blood and cerebrospinal fluid leukocytes in multiple sclerosis. *Nature communications.* 2020;11(1):1-14.

12. Hong X, Meng S, Tang D, Wang T, Ding L, Yu H, et al. Single-cell RNA sequencing reveals the expansion of cytotoxic CD4+ T lymphocytes and a landscape of immune cells in primary Sjögren's syndrome. *Frontiers in immunology.* 2021:3688.
13. Nehar-Belaid D, Hong S, Marches R, Chen G, Bolisetty M, Baisch J, et al. Mapping systemic lupus erythematosus heterogeneity at the single-cell level. *Nature immunology.* 2020;21(9):1094-106.
14. Holla P, Dizon B, Ambegaonkar AA, Rogel N, Goldschmidt E, Boddapati AK, et al. Shared transcriptional profiles of atypical B cells suggest common drivers of expansion and function in malaria, HIV, and autoimmunity. *Science Advances.* 2021;7(22):eabg8384.
15. SoRelle ED, Dai J, Reinoso-Vizcaino NM, Barry AP, Chan C, and Luftig MA. Time-resolved transcriptomes reveal diverse B cell fate trajectories in the early response to Epstein-Barr virus infection. *Cell Reports.* 2022;40(9):111286.
16. Yu G, Wang L-G, Han Y, and He Q-Y. clusterProfiler: an R package for comparing biological themes among gene clusters. *Omics: a journal of integrative biology.* 2012;16(5):284-7.
17. Price AM, Tourigny JP, Forte E, Salinas RE, Dave SS, and Luftig MA. Analysis of Epstein-Barr virus-regulated host gene expression changes through primary B-cell outgrowth reveals delayed kinetics of latent membrane protein 1-mediated NF-κB activation. *Journal of virology.* 2012;86(20):11096-106.
18. McLean CY, Bristor D, Hiller M, Clarke SL, Schaar BT, Lowe CB, et al. GREAT improves functional interpretation of cis-regulatory regions. *Nature biotechnology.* 2010;28(5):495-501.
19. McClellan MJ, Wood CD, Ojeniyi O, Cooper TJ, Kanhere A, Arvey A, et al. Modulation of enhancer looping and differential gene targeting by Epstein-Barr virus transcription factors directs cellular reprogramming. *PLoS Pathog.* 2013;9(9):e1003636.
20. Portal D, Zhou H, Zhao B, Kharchenko PV, Lowry E, Wong L, et al. Epstein–Barr virus nuclear antigen leader protein localizes to promoters and enhancers with cell transcription factors and EBNA2. *Proceedings of the National Academy of Sciences.* 2013;110(46):18537-42.
21. Shannon P, Markiel A, Ozier O, Baliga NS, Wang JT, Ramage D, et al. Cytoscape: a software environment for integrated models of biomolecular interaction networks. *Genome research.* 2003;13(11):2498-504.
22. Buenrostro JD, Giresi PG, Zaba LC, Chang HY, and Greenleaf WJ. Transposition of native chromatin for fast and sensitive epigenomic profiling of open chromatin, DNA-binding proteins and nucleosome position. *Nature methods.* 2013;10(12):1213-8.
23. Robinson JT, Thorvaldsdóttir H, Winckler W, Guttman M, Lander ES, Getz G, et al. Integrative genomics viewer. *Nature biotechnology.* 2011;29(1):24-6.

Supplementary Data

Early multiple sclerosis activity associated with TBX21^{+}CD21^{lo}CXCR3^{+} B cell expansion resembling EBV-induced phenotypes

Elliott D. SoRelle[1*#], Ellora Haukenfrers[2], Gillian Q. Horn[3], Vaibhav Jain[2], James Giarraputo[4], Karen Abramson[2], Emily Hocke[2], Laura A. Cooney[5,6], Kristina M. Harris[5], Scott S. Zamvil[5,7], Simon G. Gregory[1,2,8^*], and Micah A. Luftig[1^*]

[1] Department of Molecular Genetics and Microbiology, Duke University School of Medicine, Durham, NC 27710, USA
[2] Duke Molecular Physiology Institute, Duke University, Durham, NC 27701, USA
[3] Department of Integrative Immunobiology, Duke University School of Medicine, Durham, NC 27710, USA
[4] Yale School of Medicine, New Haven, CT 06510, USA
[5] Immune Tolerance Network, Bethesda, MD 20814, USA
[6] Department of Internal Medicine, Division of Rheumatology, University of Michigan, Ann Arbor MI 48109, USA
[7] Department of Neurology, University of California at San Francisco, San Francisco, CA 94185, USA
[8] Department of Neurology, Duke University School of Medicine, Durham, NC 27710, USA
^ Equal contributors
* Corresponding authors
[#] Current address: Department of Microbiology & Immunology, University of Michigan Medical School, Ann Arbor MI 48109, USA

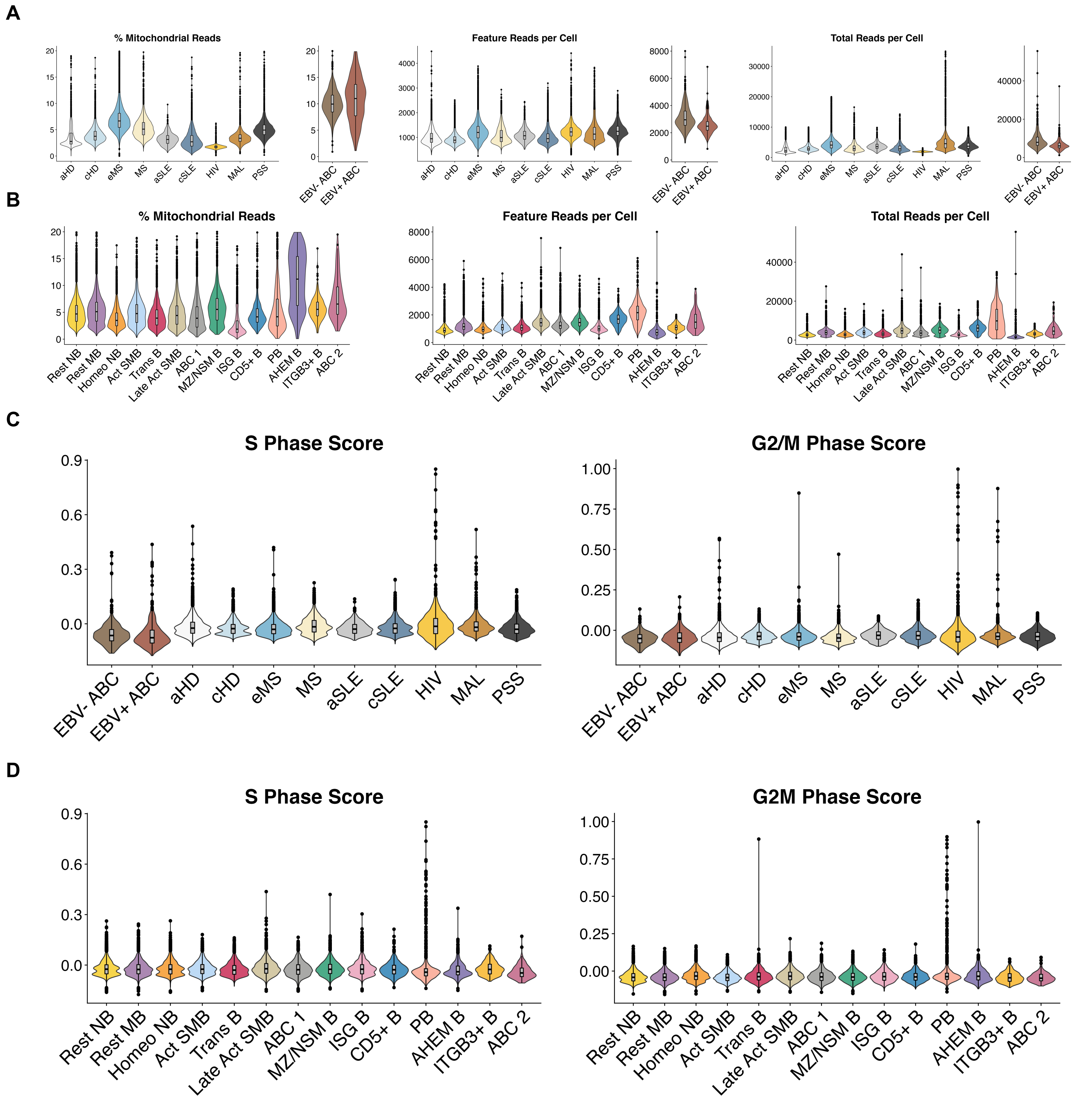

Figure S1. QC characterization of B cells by dataset and annotated B subsets.
A) Mitochondrial gene fraction, average feature mRNA per cell, and average total mRNA per cell by dataset.
B) Mitochondrial gene fraction, average feature mRNA per cell, and average total mRNA per cell by annotated B cell subset.
C) Cell cycle scoring (S phase, G_2/M phase) of B cells by dataset.
D) Cell cycle scoring (S phase, G_2/M phase) by annotated B cell subset.

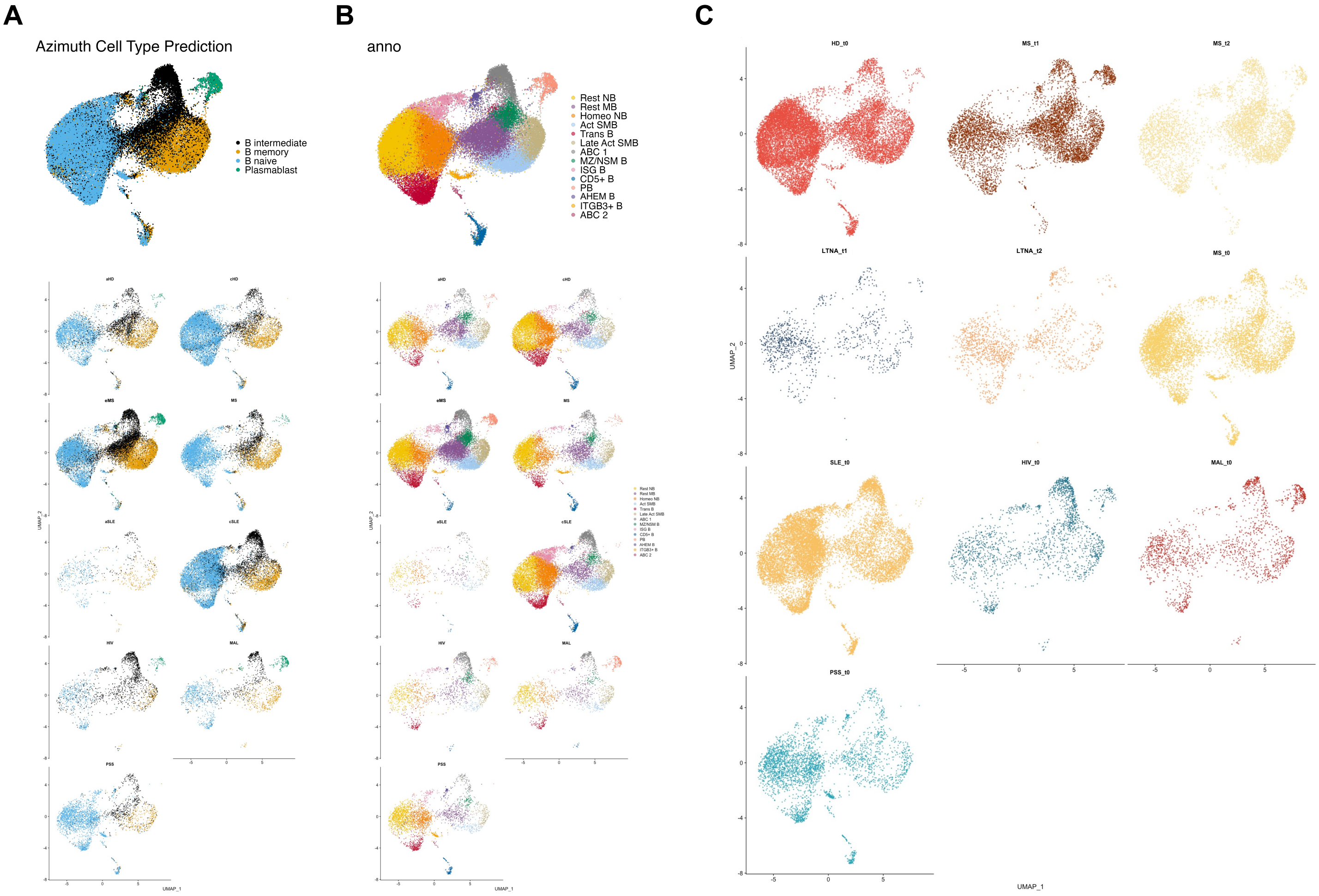

Figure S2. Integrated B cell type prediction and annotation by dataset and outcome.

A) Cell type prediction from Azimuth (74) used for initial B cell isolation. Naïve B cells (light blue), intermediate B cells (black), memory B cells (gold), and plasmablasts (green) were included from all analyzed datasets for integrated analysis. Predicted cell type UMAPs are presented for all integrated datasets and each dataset individually.

B) High resolution annotation of B cell subsets identified from unbiased hierarchical clustering methods. Annotations were assigned based on Azimuth reference cell type scores and cluster-resolved differential gene expression in conjunction with literature-based curation. B subset annotation UMAPs are presented for all integrated datasets and each dataset individually.

C) UMAP representation of cells by time-resolved outcome for each dataset. Early MS (eMS) B cells are split by time (t1, t2) and outcome (SMSA, LTNA). Cells from all publicly available datasets collected at single timepoints are labeled as t0 samples (e.g., HD_t0=healthy donors; MS_t0=MS).

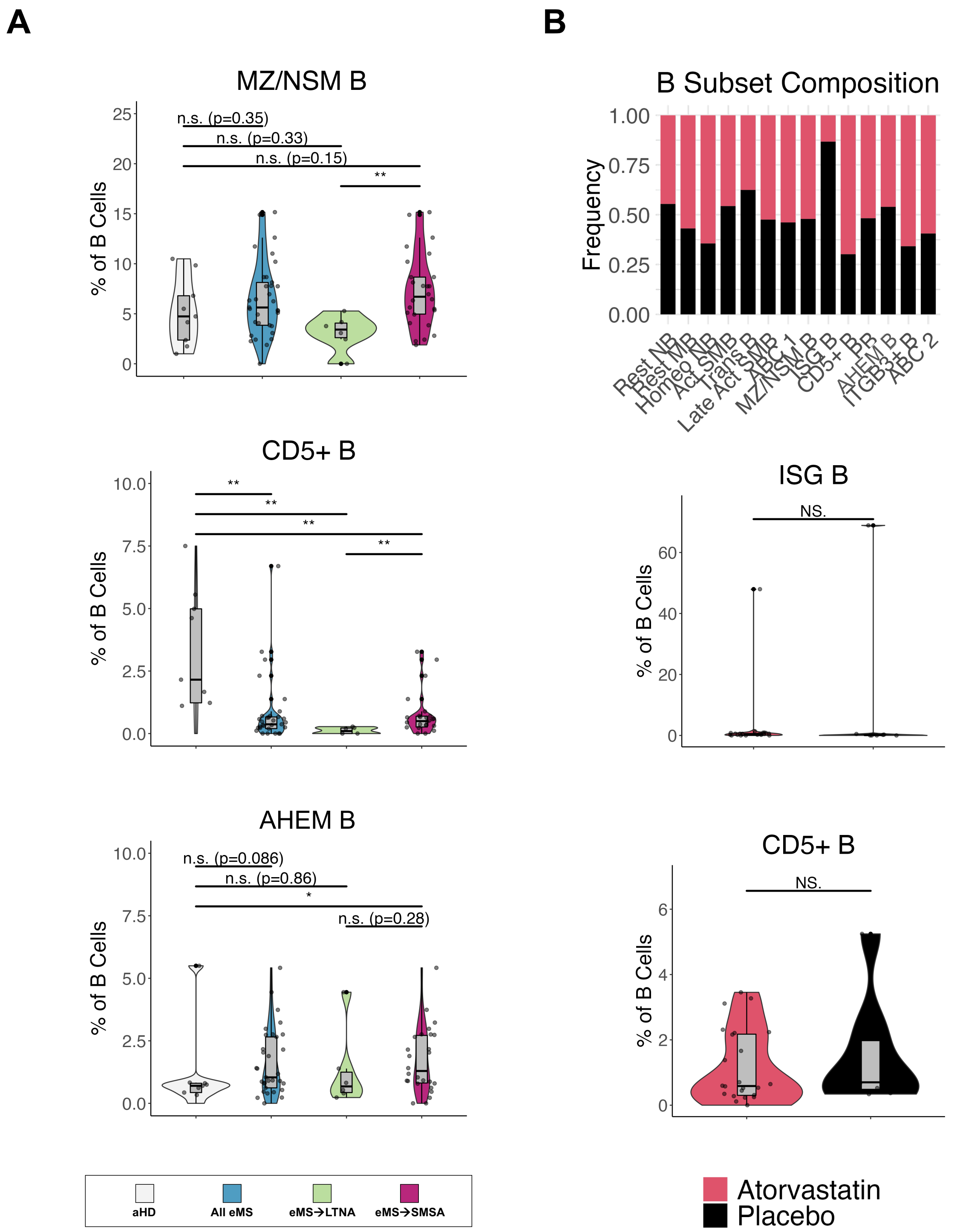

Figure S3. Cell frequencies in healthy adults and eMS cohort across other B subsets of interest.
A) Fractions of Marginal Zone + Non-Switched Memory (MZ/NSM) B cells, $CD5^{+}$ B cells (CD5+ B), and B cells with elevated autophagy, ATP hydrolysis, and epigenetic modifier expression (AHEM B) in healthy adults (eggshell white), all people with eMS (medium blue), people with eMS identified as long-term no evidence of activity (LTNA; seafoam green), and people with eMS exhibiting subsequent MS activity (SMSA; magenta). Statistical comparisons were made by two-sided Wilcoxon rank sum test. P values are provided for non-significant comparisons (n.s.) and denoted with asterisks for significance (* p<0.05; ** p<0.01; *** p<0.001).
B) Sample size-normalized B subset frequency stratified by treatment group (atorvastatin (red) versus placebo (black). Statistical comparisons of the most variable subsets by treatment group did not identify significant differences.

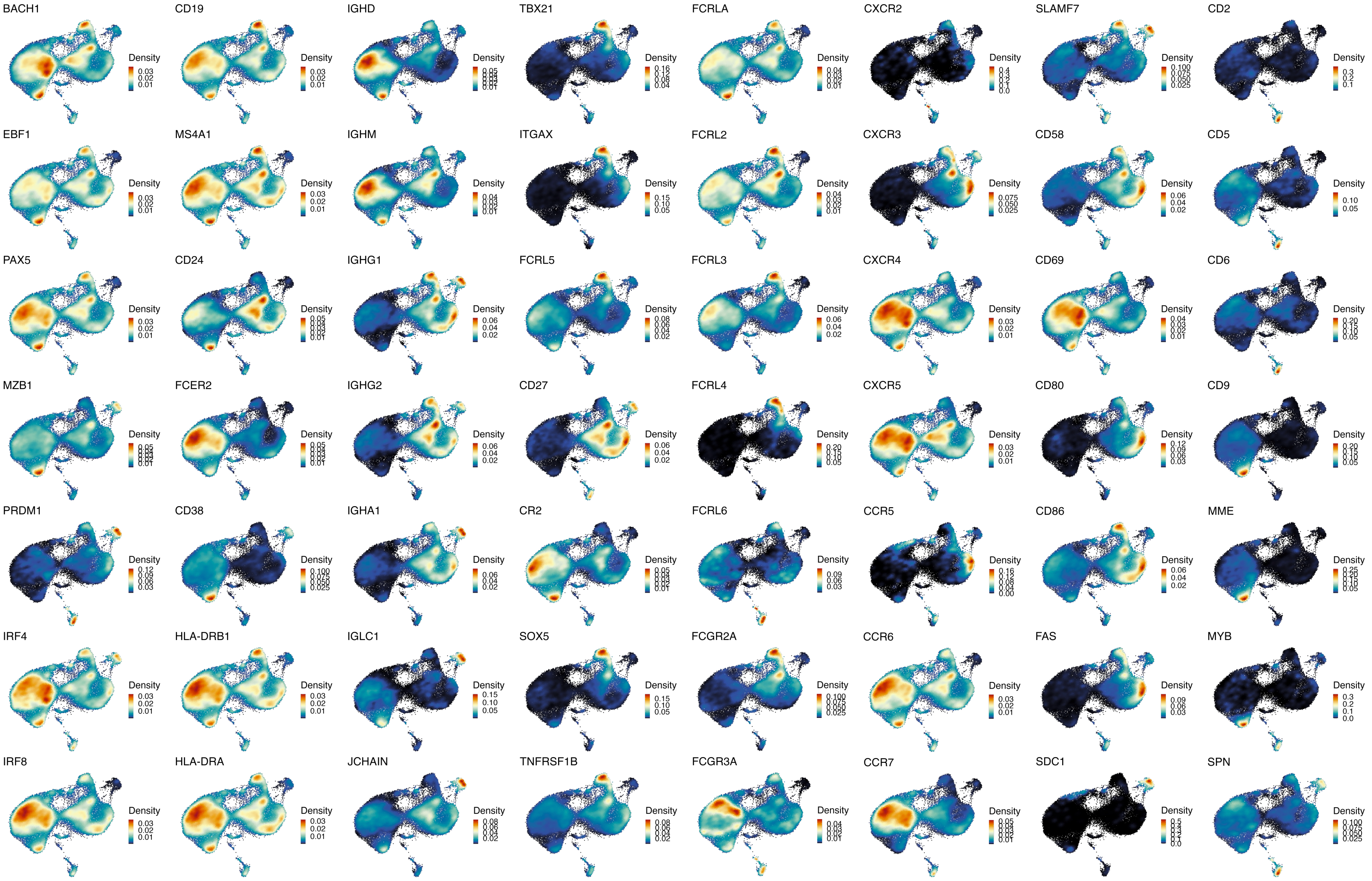

Figure S4. UMAP representation of genes used for B cell phenotyping.
Gene expression in B cells from all integrated datasets. Expression is visualized as weighted kernel density estimation generated with the Nebulosa package in R (148).

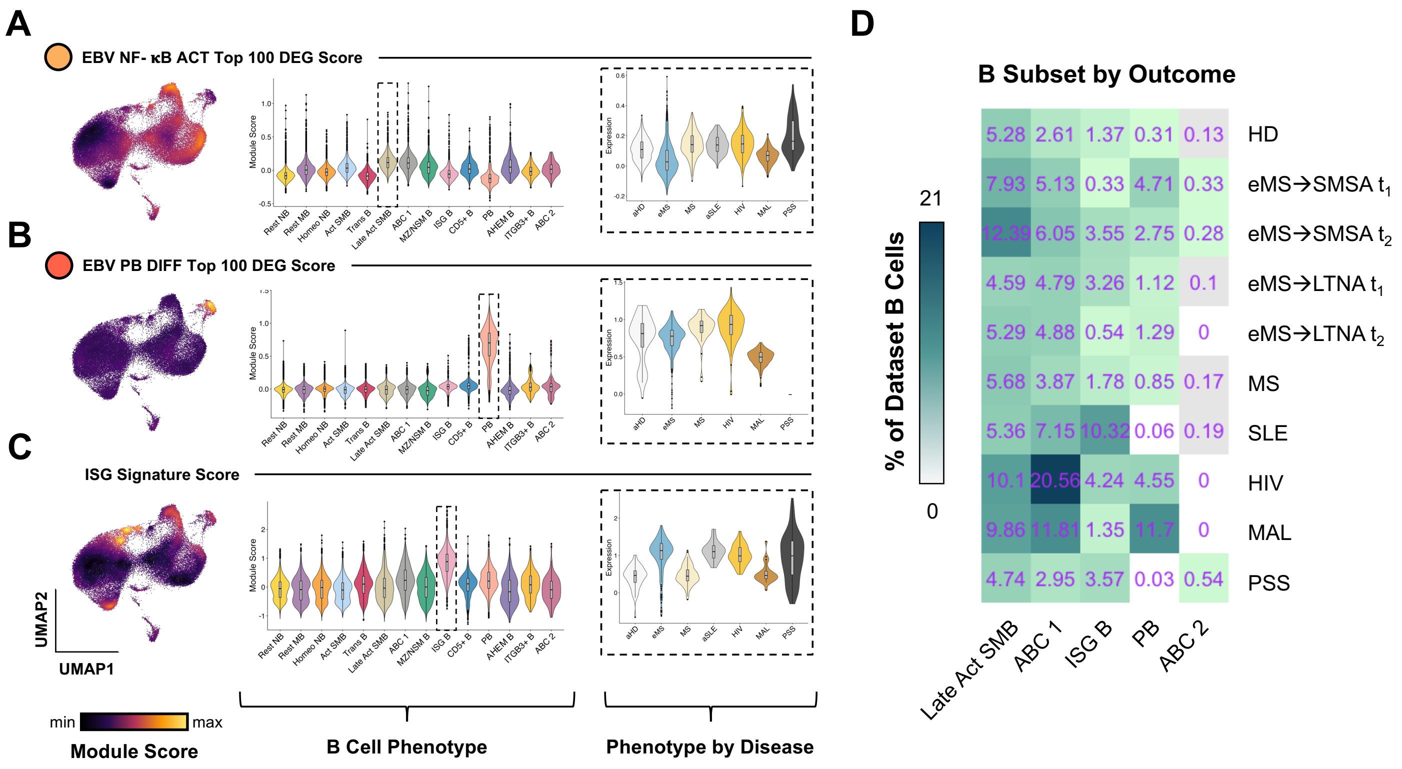

Figure S5. Module scores for additional EBV-associated phenotypes from *in vitro* infection scRNA-seq data.

A) UMAP and violin plot representation of the *in vitro* EBV+ NF-κB activation gene signature across all integrated B cells from disease and healthy control datasets. Signature module scores were calculated from the top 100 DEGs in EBV+ NF-κB activated cells versus resting B cells.

B) UMAP and violin plot representation of the *in vitro* EBV+ PB (differentiation) gene signature across all integrated B cells from disease and healthy control datasets. Signature module scores were calculated from the top 100 DEGs in EBV+ PBs versus resting B cells.

C) UMAP and violin plot representation of a curated interferon-stimulated gene (ISG) signature described in the context of SLE (72).

D) Select B cell subset frequencies in disease cohorts and healthy individuals. Subset frequencies for the eMS cohort reported herein are split by collection timepoint (t_1, t_2) and outcome (SMSA, LTNA).

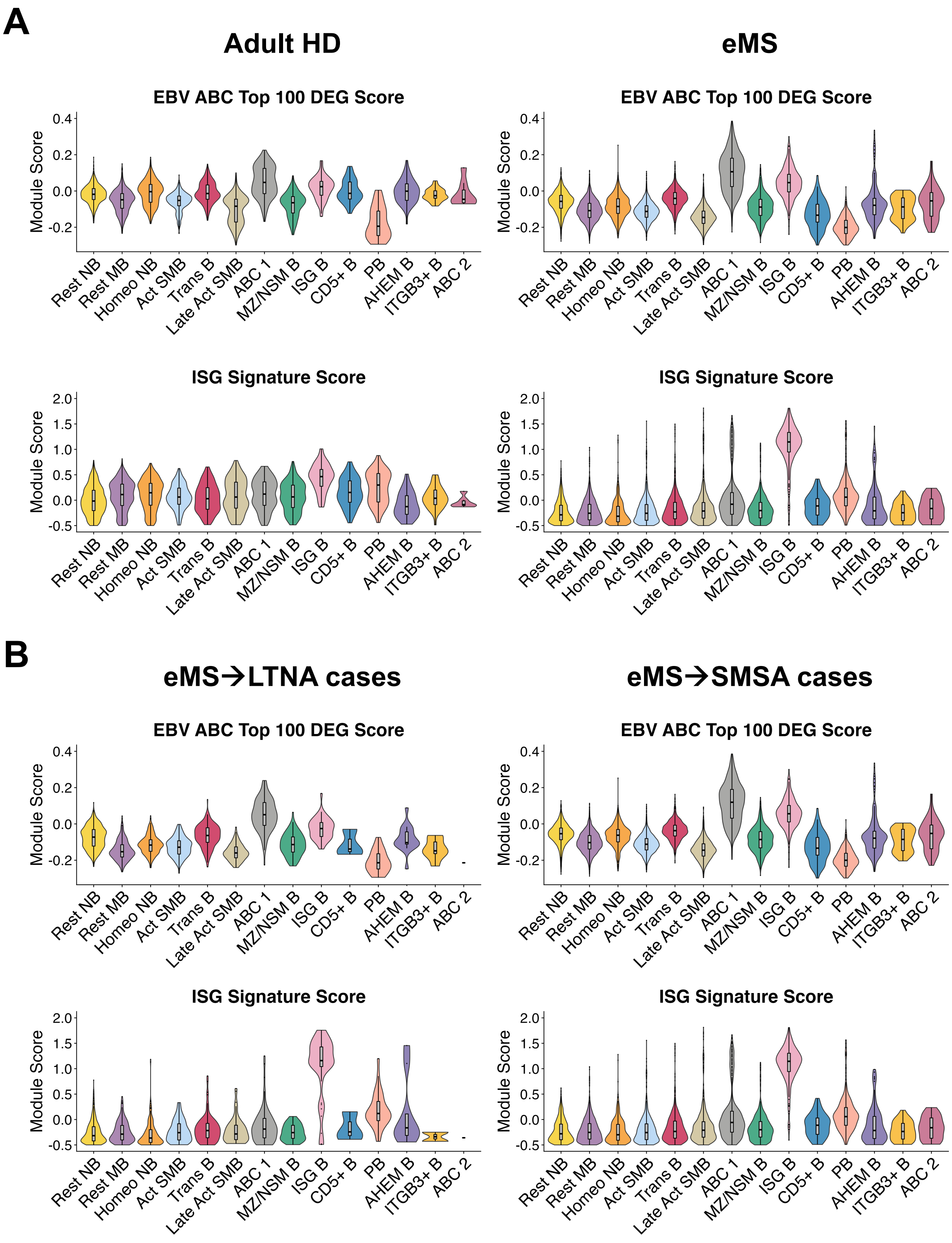

Figure S6. EBV⁺ ABC and ISG signature scores by B subset in healthy adults and early MS cohort.
A) EBV⁺ ABC Top 100 DEG scoring across B cell subsets in healthy adults and people with early MS (top row). Curated ISG signature scoring across B cell subsets in healthy adults and people with early MS (bottom row).
B) EBV⁺ ABC Top 100 DEG scoring across B cell subsets in outcome-stratified early MS cohort (top row). Curated ISG signature scoring across B cell subsets in outcome-stratified early MS cohort (bottom row).

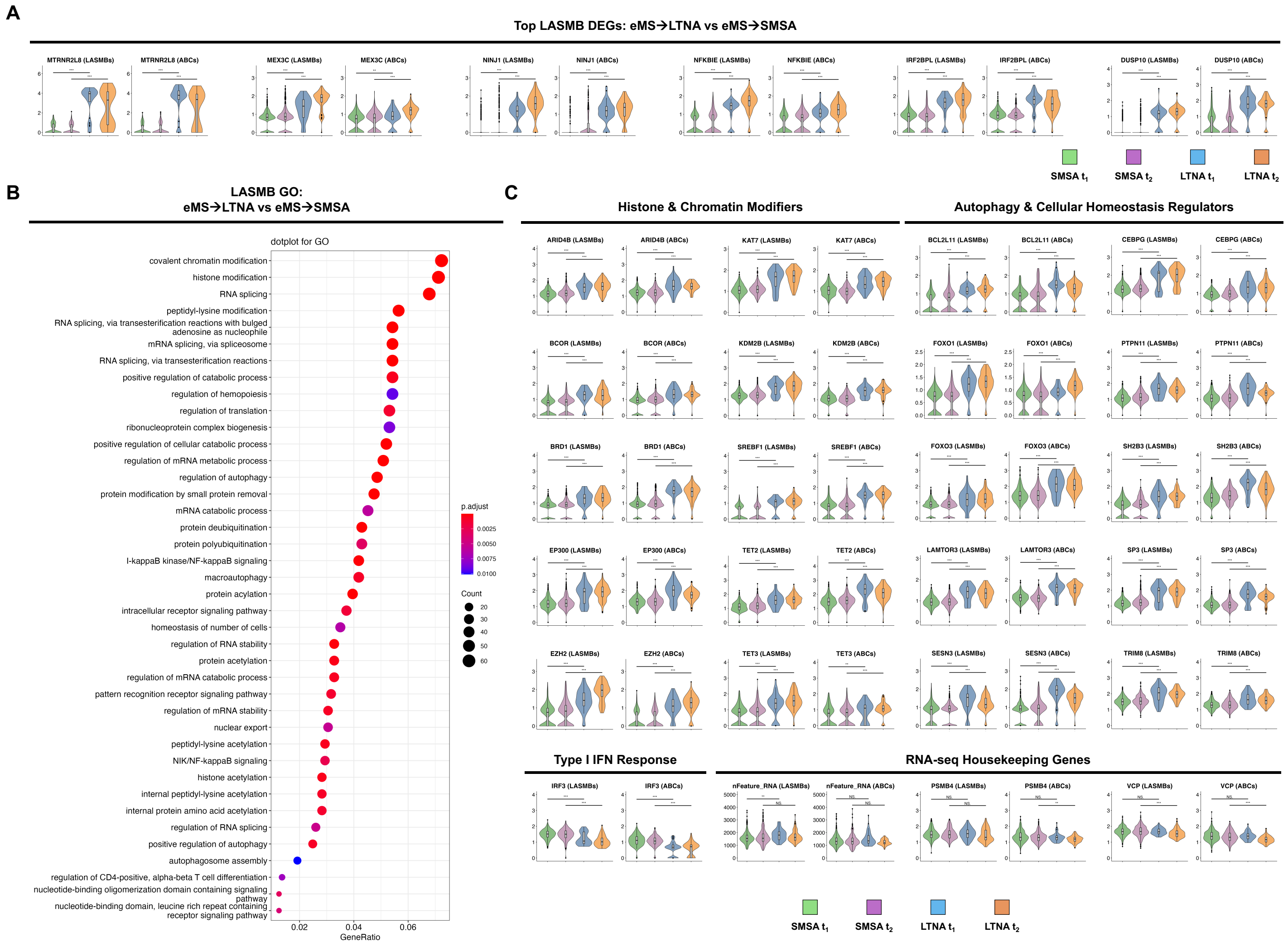

Figure S7. Outcome-stratified differential gene expression in late activated switched memory B cells from people with early MS.

A) Selected top differentially expressed genes (DEGs) elevated in Late Act SMB (LASMB) from eMS→LTNA versus eMS→MS outcomes. Expression is also presented for the ABC 1 and ABC 2 subsets (ABCs). Statistical comparisons were made by two-sided Wilcoxon rank sum test. P values are provided for non-significant comparisons (n.s.) and denoted with asterisks for significance (* p<0.05; ** p<0.01; *** p<0.001).

B) Gene ontology (GO) biological process enrichment result for genes significantly elevated in LASMB from eMS→LTNA versus eMS→SMSA outcomes. Dot size represents the number of DEGs associated with a significantly enriched GO term. Dot color denotes the Bonferroni-corrected p value for enriched GO terms.

C) Outcome-stratified expression of selected DEGs associated with significantly enriched GO terms. Expression is presented for LASMB and ABC subsets. Several enriched terms and DEGs elevated in eMS→LTNA versus eMS→SMSA (e.g., autophagy, cellular homeostasis) were associated with resolution of inflammatory responses. Accordingly, expression of the Type I interferon response mediator *IRF3* was significantly attenuated in eMS→LTNA versus eMS→SMSA (bottom row). Expression of total mRNA features and RNA-seq housekeeping genes was also evaluated to confirm that outcome-stratified DEGs in LASMBs and ABCs were not technical artifacts. Statistical comparisons were performed and denoted as described in S6A.

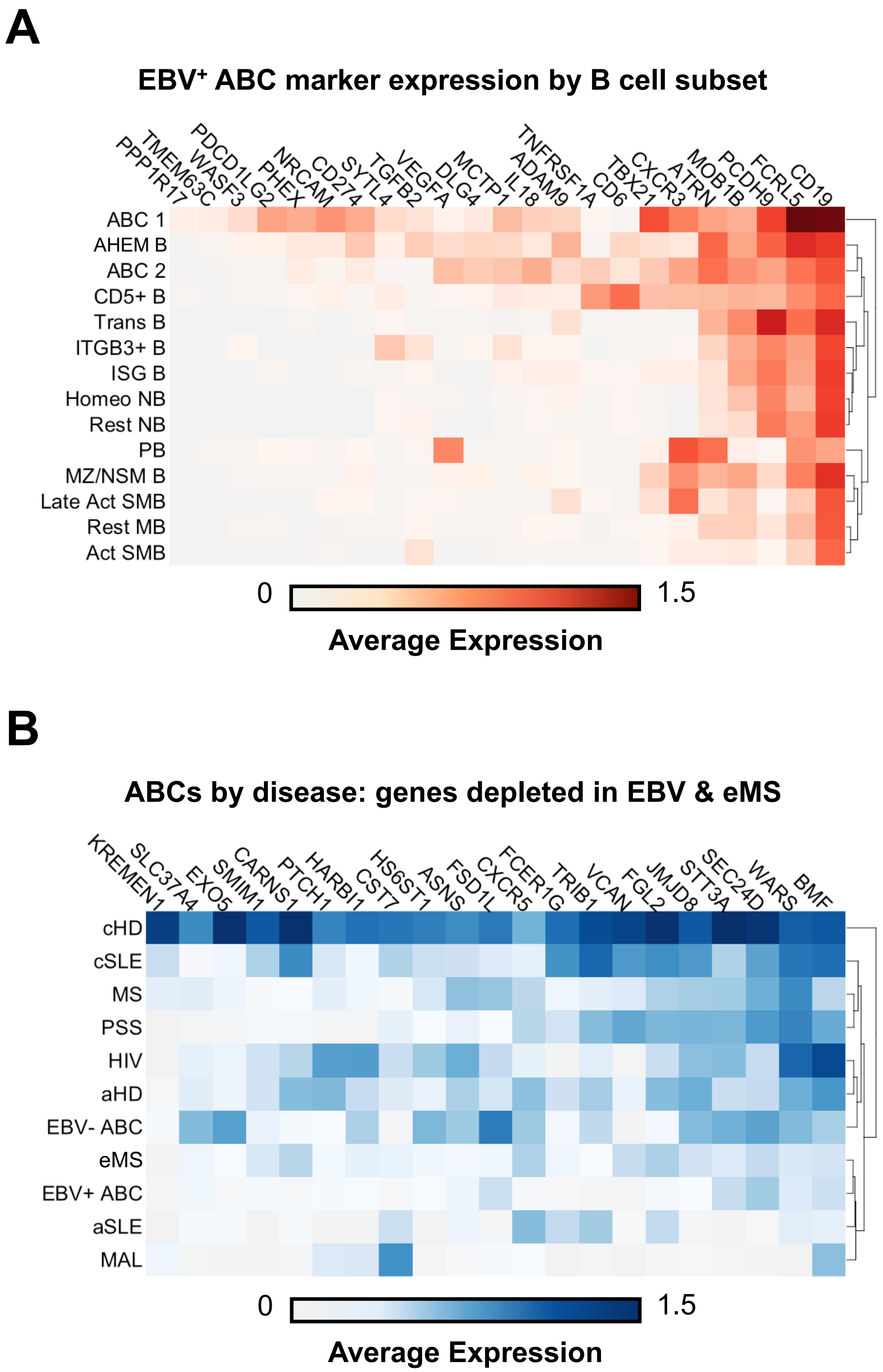

Figure S8. Average EBV+ ABC marker expression by B cell subset and disease dataset.
A) Average expression of markers elevated in both EBV+ ABCs *in vitro* and ABCs from patients with eMS hierarchically ordered by B subset.
B) Average expression of markers depleted in EBV+ ABCs and *in vitro* and ABCs from people with eMS hierarchically ordered by dataset.

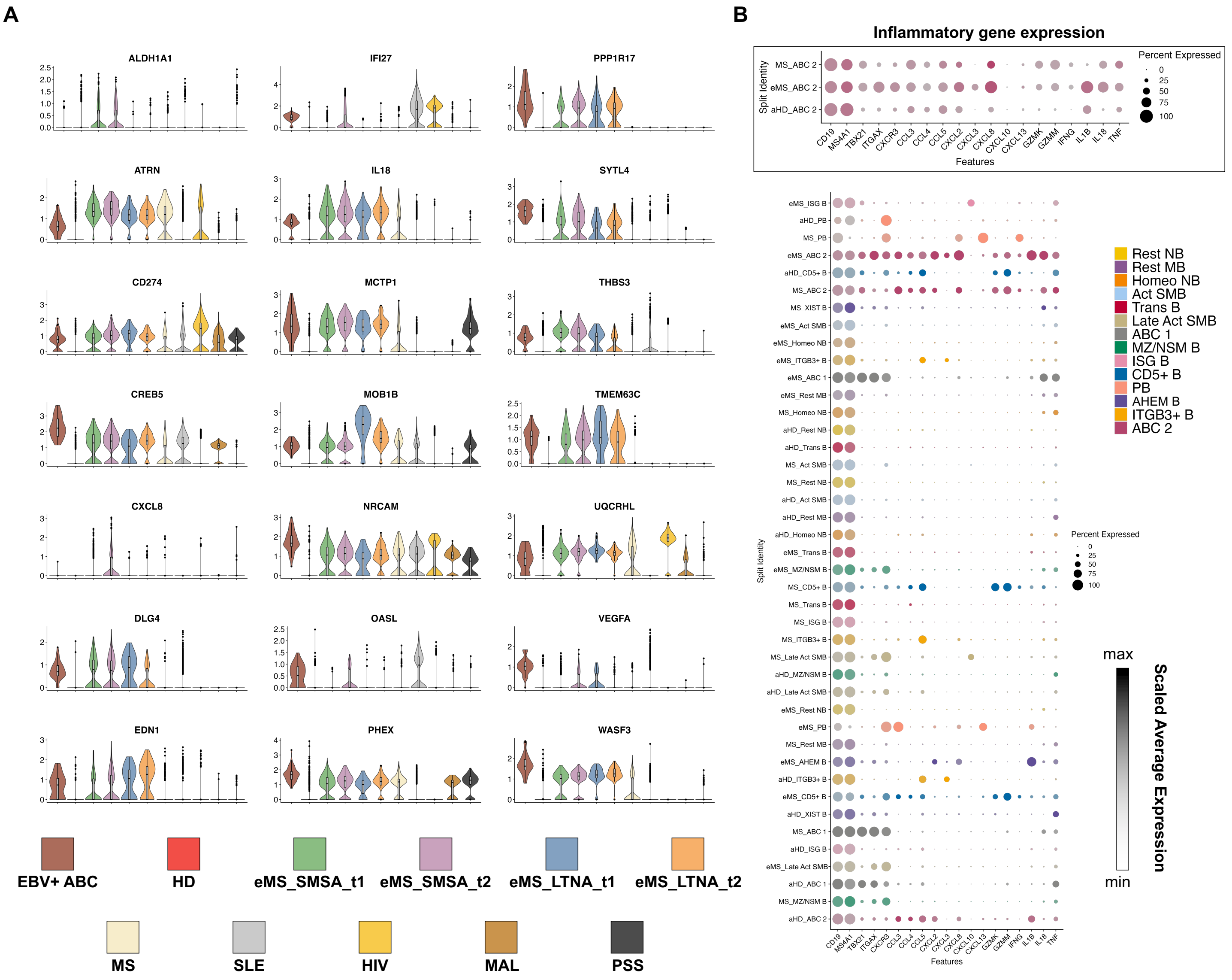

Figure S9. Dataset and outcome-stratified expression of select EBV⁺ ABC signature and inflammatory genes.

A) Violin plots of EBV^{+} ABC markers expression across disease datasets. Samples from the longitudinal eMS cohort are split by outcome and timepoint. Expression in *de novo*-infected ABCs from *in vitro* studies is provided for comparison.

B) Dot plots of inflammatory gene expression in B subsets from patients with eMS, MS, and healthy controls. Average expression (unscaled) in ABC 2 subset from longitudinal eMS cohort, publicly available peripheral B cell data from people with MS, and healthy controls from multiple publicly available datasets (top panel). Dot size represents the percent of ABC 2 cells from each context expressing a given gene. Scaled average expression of inflammatory genes across all B subsets in people with eMS, MS, and healthy controls (bottom panel). Darker hues represent higher expression levels within a subset.

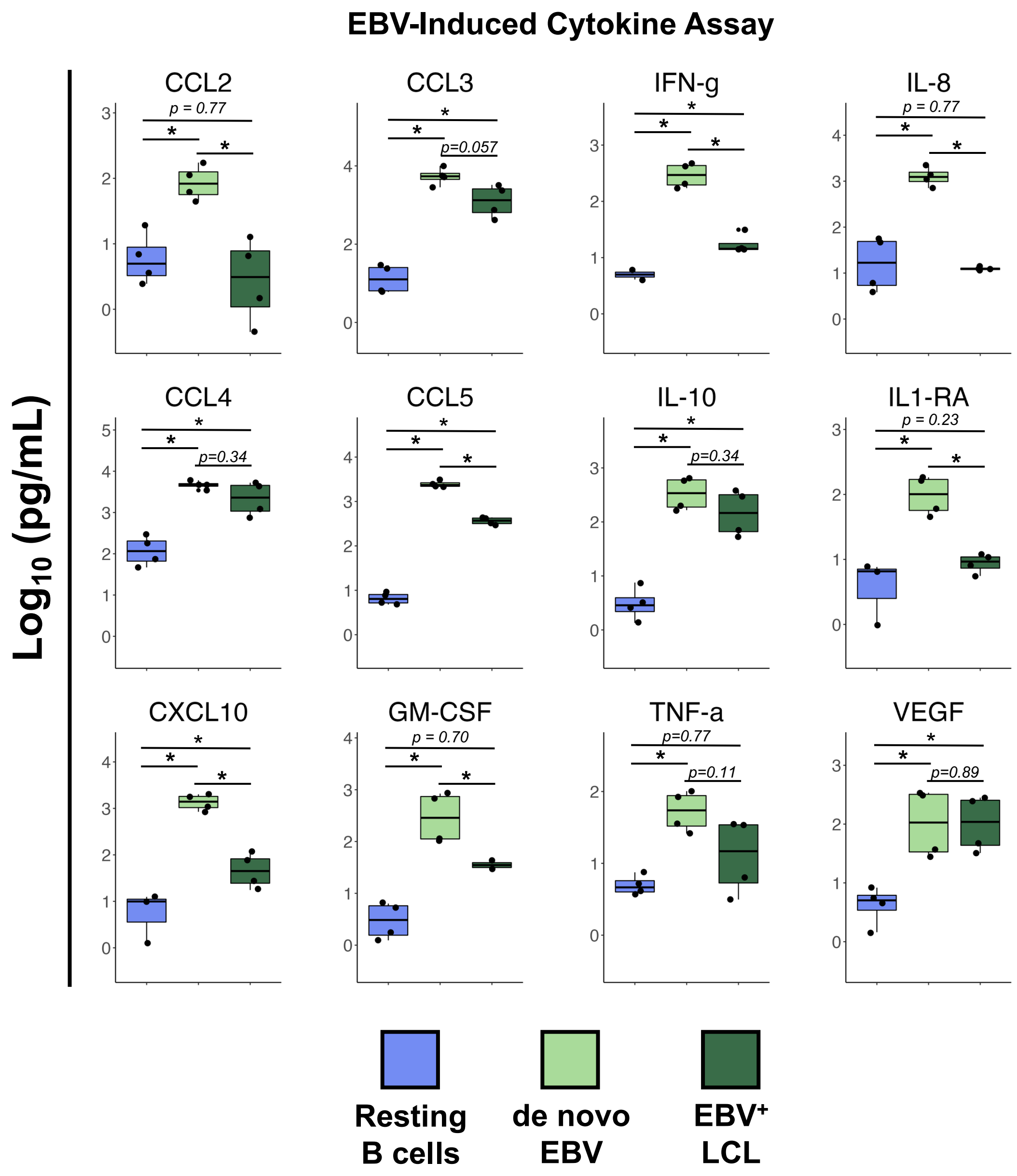

Figure S10. Cytokine secretion by resting, EBV *de novo*-infected, and EBV-immortalized cells (LCLs). Analysis of cytokine secretion induced by *de novo* (light green) and latent (dark green) EBV infection versus resting B cells (blue). Boxplots represent median and first and third quartile concentrations (pg/mL) on logarithmic scale (n=4; two technical replicates from each of two biological replicates). Statistical significance was evaluated by two-sided Kolmogorov-Smirnov Test (* $p < 0.05$).

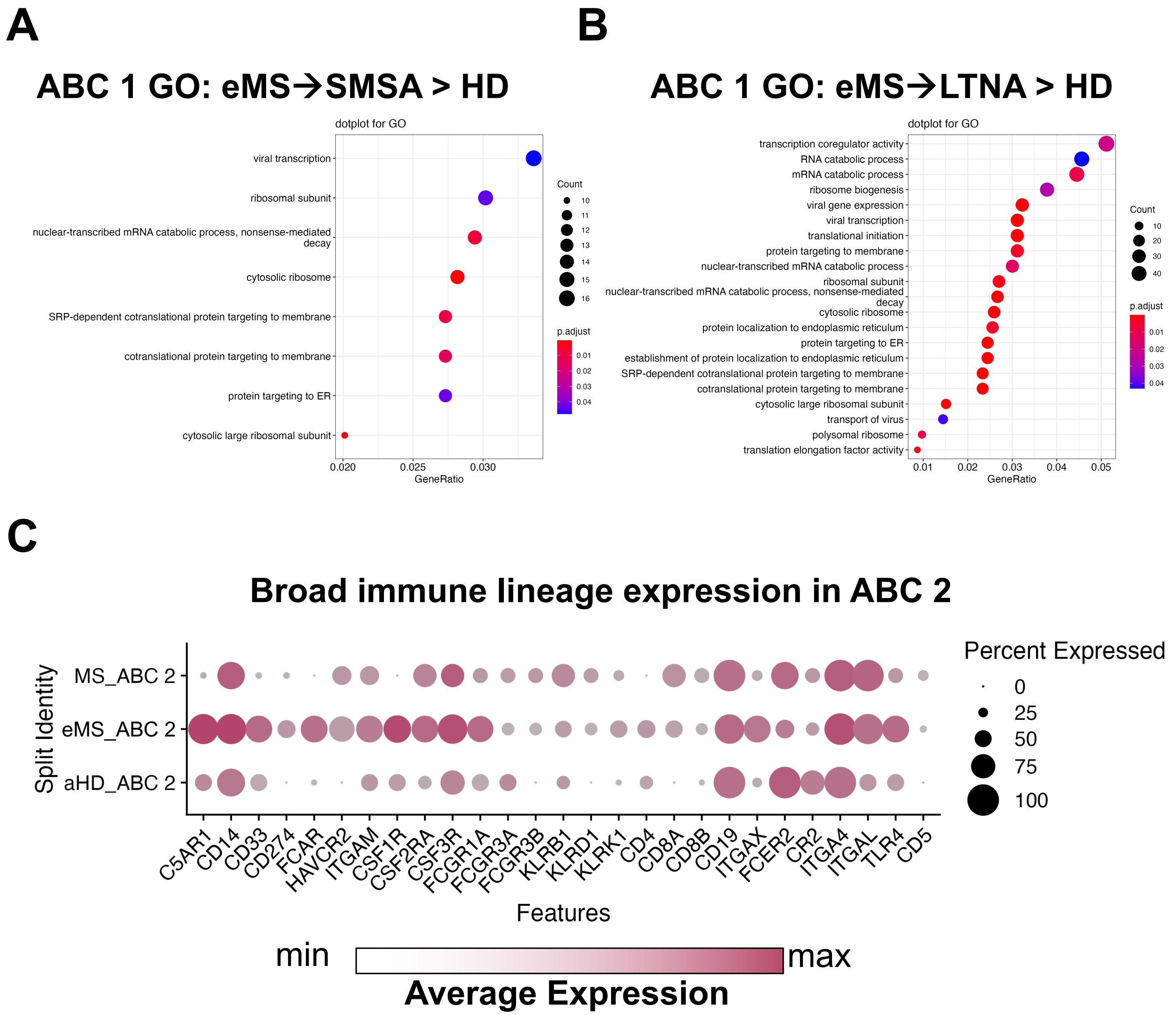

Figure S11. Gene ontology enrichment in ABCs for eMS outcomes versus healthy adults.

A) Gene ontology (GO) enrichment in ABC 1 subset from eMS→SMSA outcomes versus healthy adults. Dot size represents the number of DEGs associated with a significantly enriched GO term. Dot color denotes the Bonferroni-corrected p value for enriched GO terms.

B) Gene ontology (GO) enrichment in ABC 1 subset from eMS→LTNA outcomes versus healthy adults. Dot size represents the number of DEGs associated with a significantly enriched GO term. Dot color denotes the Bonferroni-corrected p value for enriched GO terms.

C) Broad immune lineage gene expression in ABC 2 subset from eMS, MS, and healthy controls.

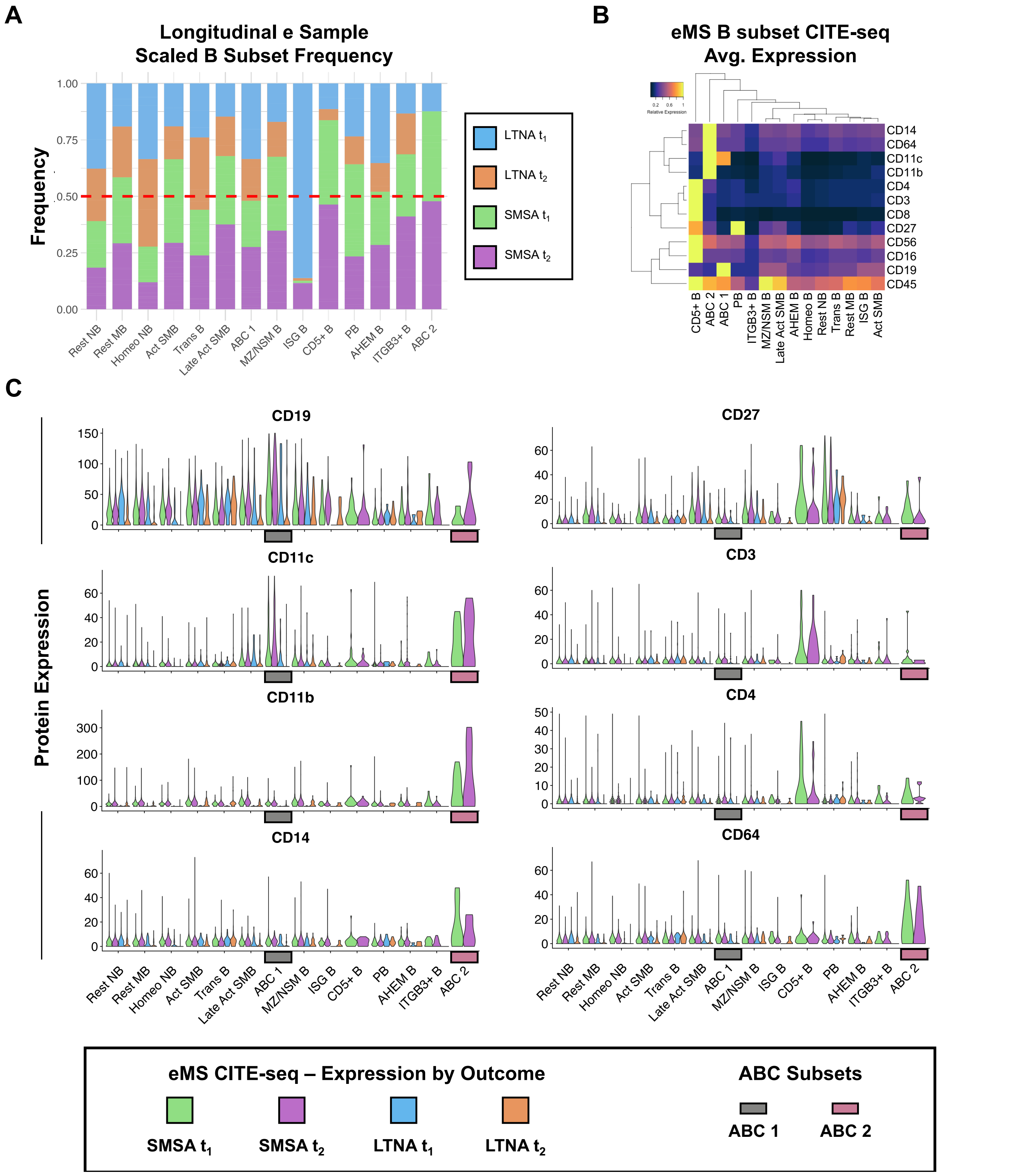

Figure S12. CITE-seq protein biomarker expression data in eMS dataset.

A) Sample size-normalized B subset frequency in eMS stratified by timepoint and patient outcome (SMSA vs LTNA). Red dashed line denotes equivalent normalized subset frequency by outcome (50%).

B) Relative average surface protein expression in B subsets from eMS assayed via CITE-seq. B subsets and surface markers are hierarchically ordered by expression similarity.

C) Surface protein expression stratified by B subset and time-resolved stratified outcome in eMS cohort.

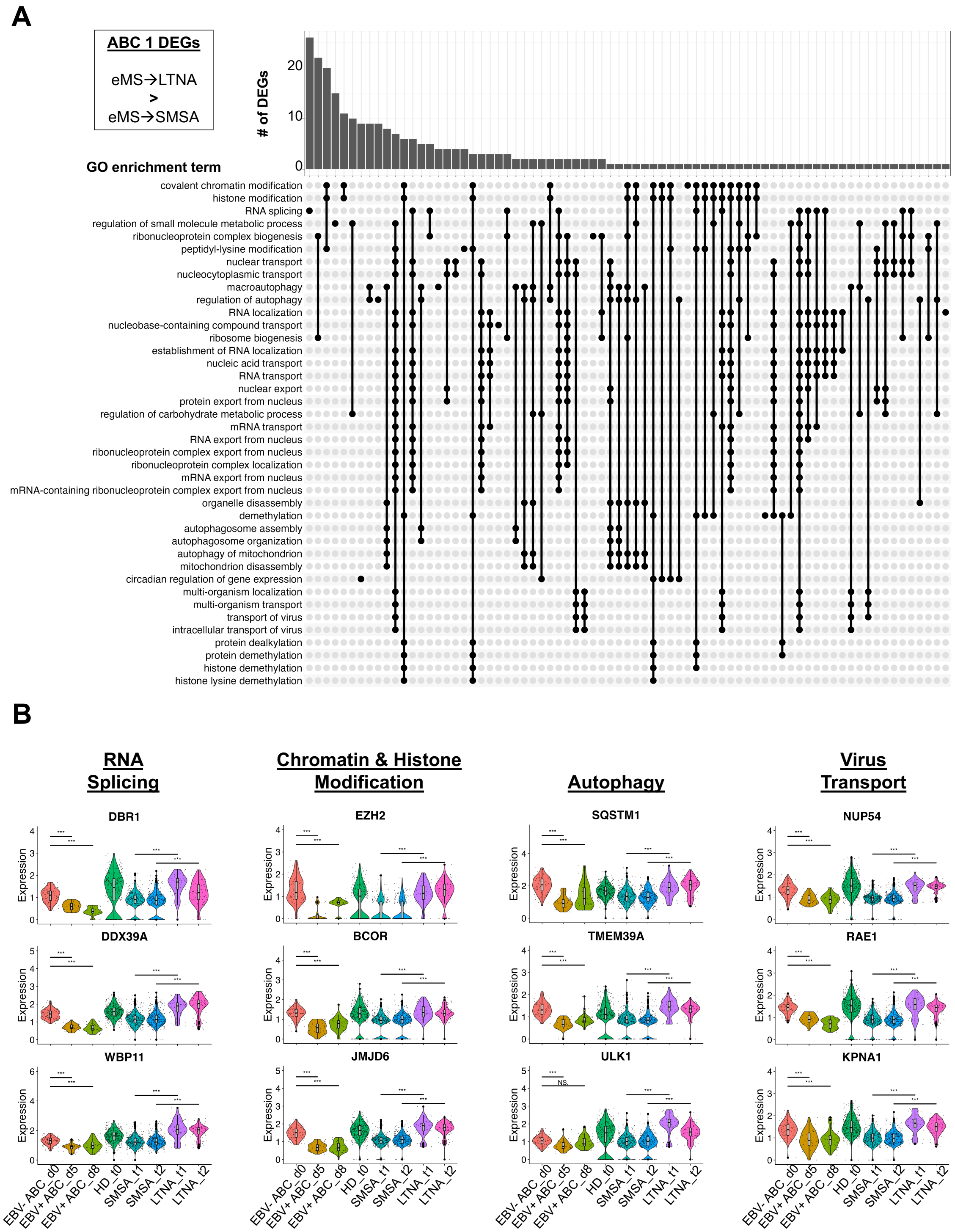

Figure S13. Gene ontology and differential expression in ABC 1 subset from eMS→LTNA versus eMS→SMSA outcomes.

A) Upset plot of significant gene ontology (GO) terms and associated number of genes enriched in eMS→LTNA versus eMS→SMSA samples for the ABC 1 subset. Histogram depicts the number of differentially expressed genes (DEGs) associated with one or more enriched GO terms indicated by upset diagram.

B) Expression of selected DEGs elevated in eMS→LTNA versus eMS→SMSA ABC 1 subset. Gene expression within time-resolved *in vitro* EBV infection of ABCs is also depicted. Statistical comparisons are presented for ABCs before and after *de novo* EBV infection *in vitro* as well as time-matched samples from LTNA and SMSA outcomes. Statistical comparisons were made by two-sided Wilcoxon rank sum test. P values are provided for non-significant comparisons (n.s.) and denoted with asterisks for significance (* p<0.05; ** p<0.01; *** p<0.001).

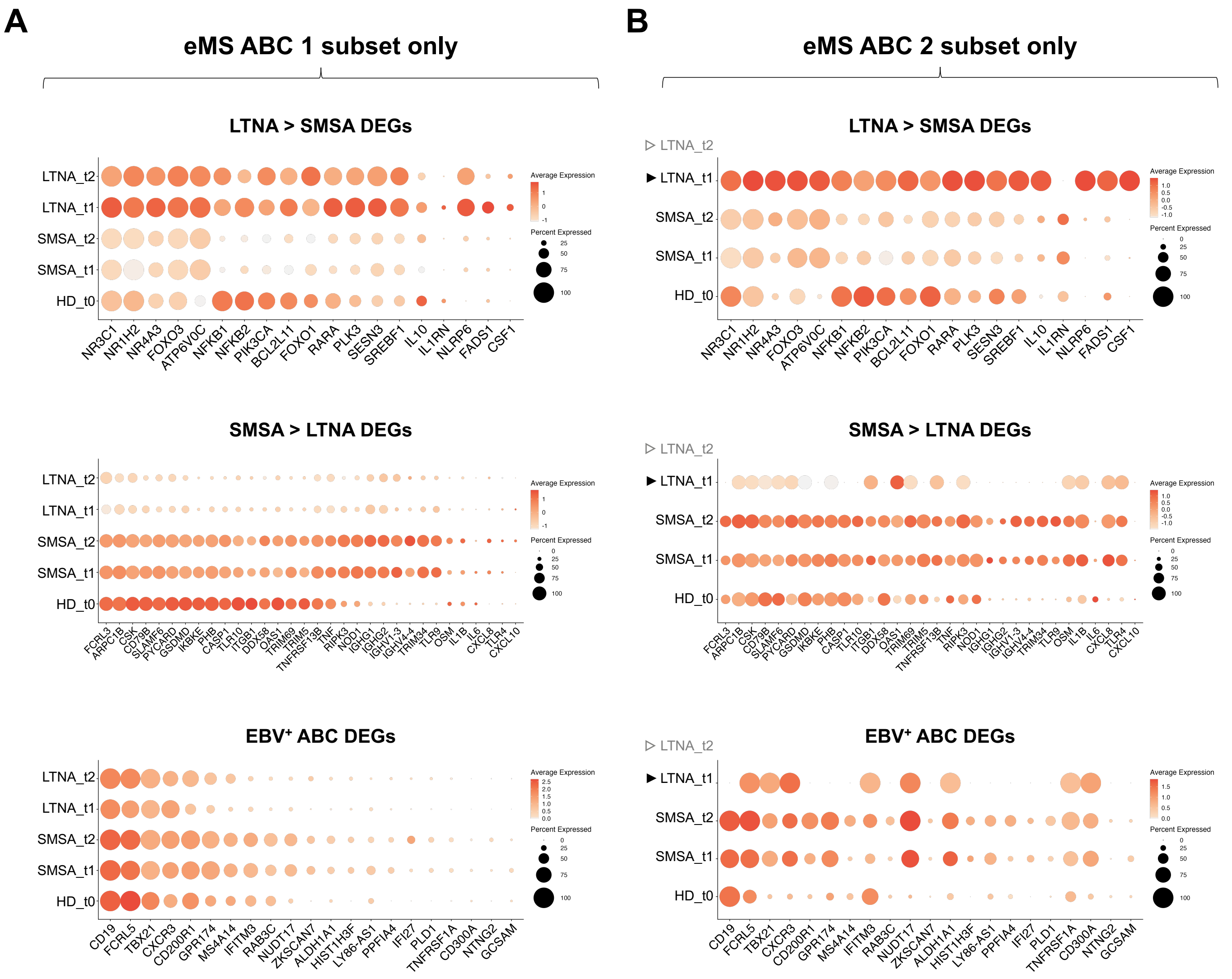

Figure S14. Differential gene expression in ABC subsets in people with eMS and healthy adults stratified by timepoint and outcome.

A) Selected differentially expressed genes in ABC 1 subset. Higher expression of genes from enriched GO terms related to autophagy and inflammatory response resolution in LTNA ABC 1 versus SMSA ABC 1 (top row). Higher expression of genes from enriched GO terms related to B cell activation, innate responses, and antiviral sensing in SMSA ABC 1 versus LTNA ABC 1 (middle row). EBV$^+$ ABC signature genes with higher expression in SMSA ABC 1 versus LTNA ABC 1 (bottom row). Dot size represents the percent of ABCs expressing a gene. Dot color denotes scaled average gene expression.

B) Selected differentially expressed genes in ABC 2 subset. No cells were detected in ABC 2 for LTNA samples at t2 (open triangle), and gene expression measurements for ABC 2 in LTNA samples at t1 were derived from one cell (filled triangle). Higher expression of genes from enriched GO terms related to autophagy and inflammatory response resolution in LTNA ABC 2 versus SMSA ABC 2 (top row). Higher expression of genes from enriched GO terms related to B cell activation, innate responses, and antiviral sensing in SMSA ABC 2 versus LTNA ABC 2 (middle row). EBV$^+$ ABC signature genes with higher expression in SMSA ABC 2 versus LTNA ABC 2 (bottom row). Dot size represents the percent of ABCs expressing a gene. Dot color denotes scaled average gene expression.

eMS ABC 2 vs ABC 1 GO

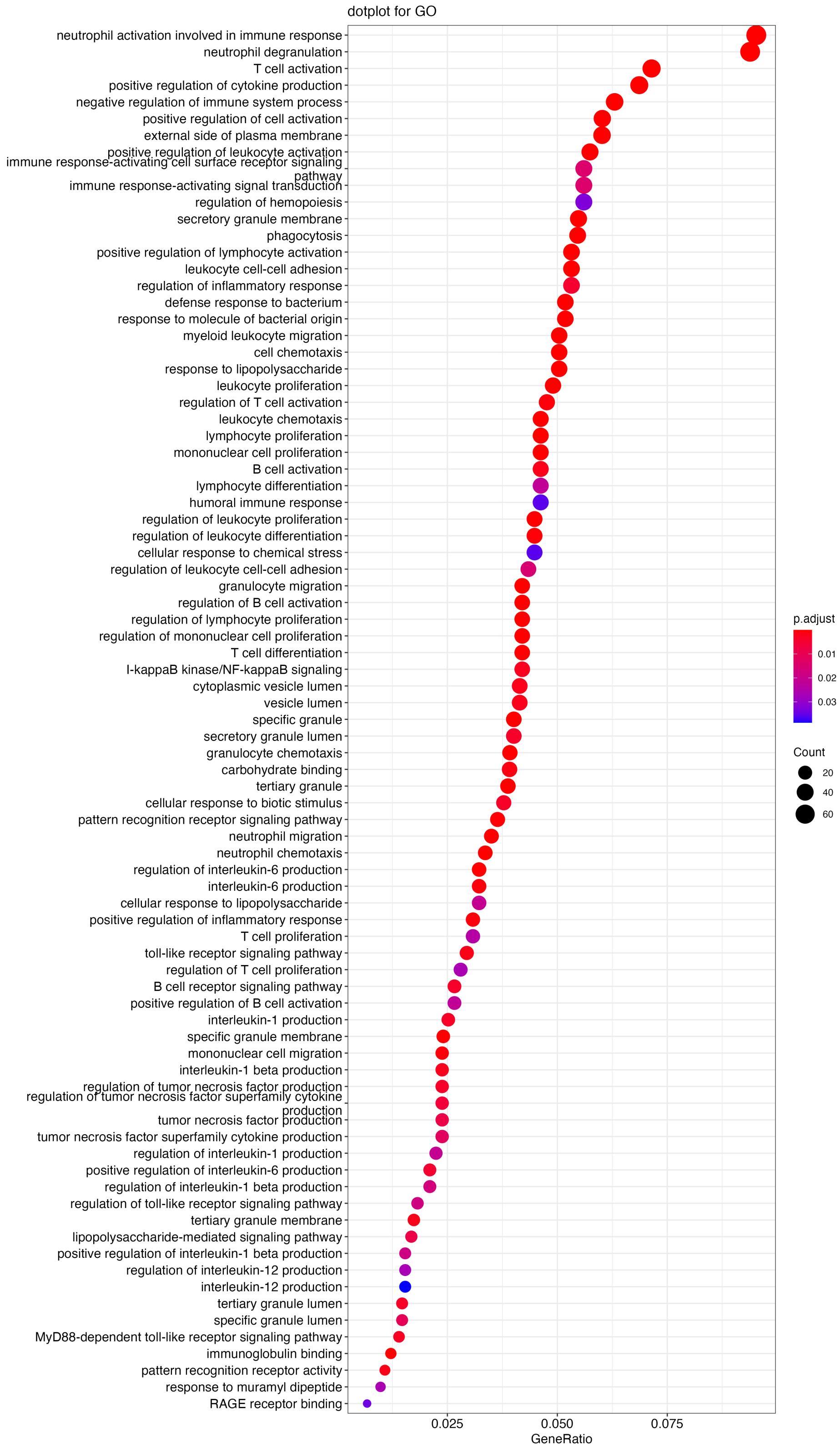

Figure S15. Gene ontology (GO) enrichment of ABC 2 versus ABC 1 subsets in people with eMS.

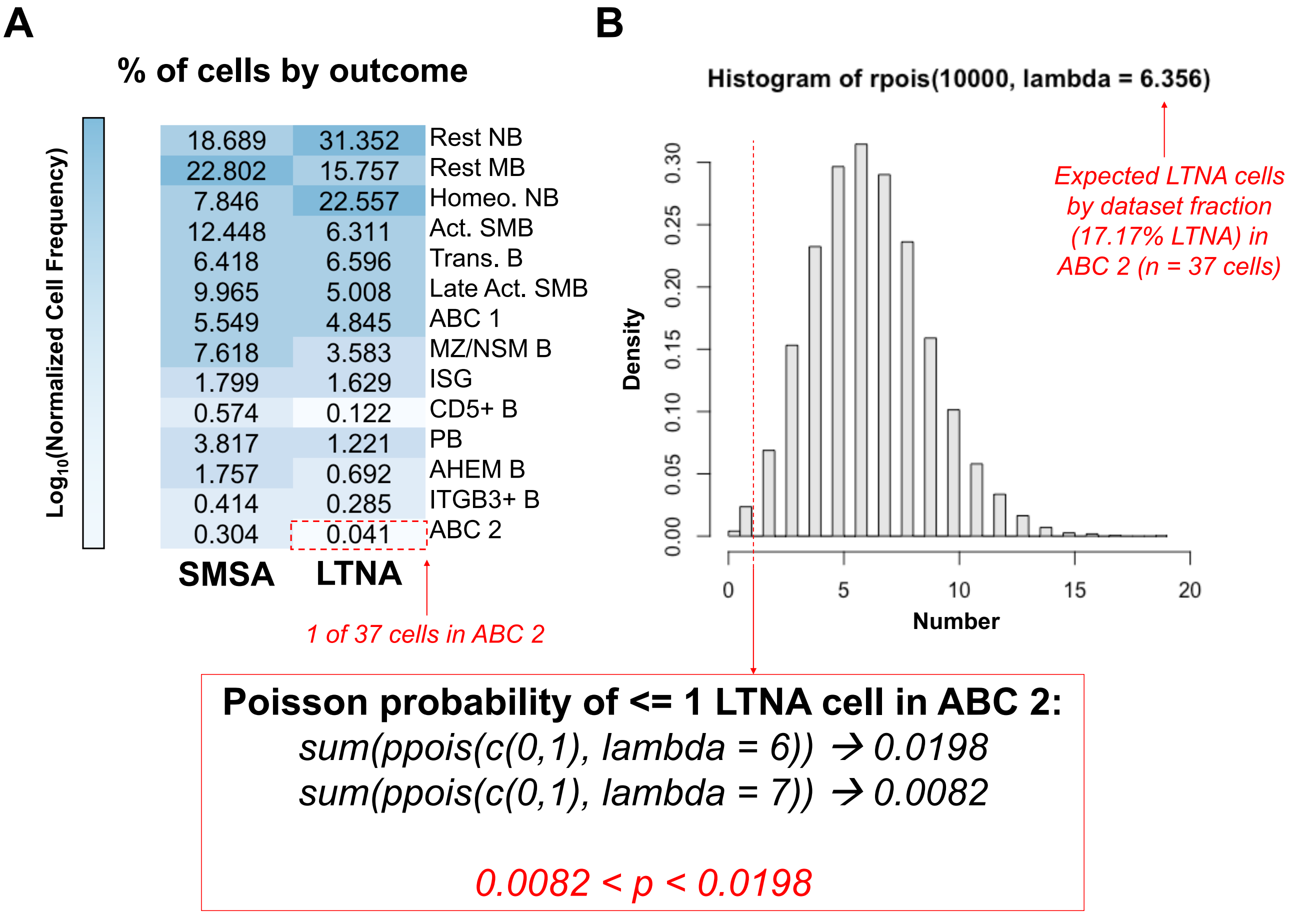

Figure S16. Poisson probability calculations for ABC 2 phenotype frequency in eMS→SMSA versus eMS→LTNA outcomes.

A) Heatmap of sample size-normalized B subset frequencies in people with eMS stratified by outcome. One out of 37 ABC 2 cells measured from people with eMS was derived from LTNA samples.

B) Poisson simulation outcome (10,000 iterations) for random sampling with lambda equal to the expected number of LTNA-derived cells based on single-cell library sample sizes (λ=6.356=37 ABC 2 cells x 17.17% all eMS cells derived from LTNAs). Dashed red line denotes the actual number of LTNA-derived cells observed (1). Poisson probability range for measuring the observed number of LTNA-derived ABC 2 cells or fewer by random chance ($0.0082 < p < 0.0198$).

A

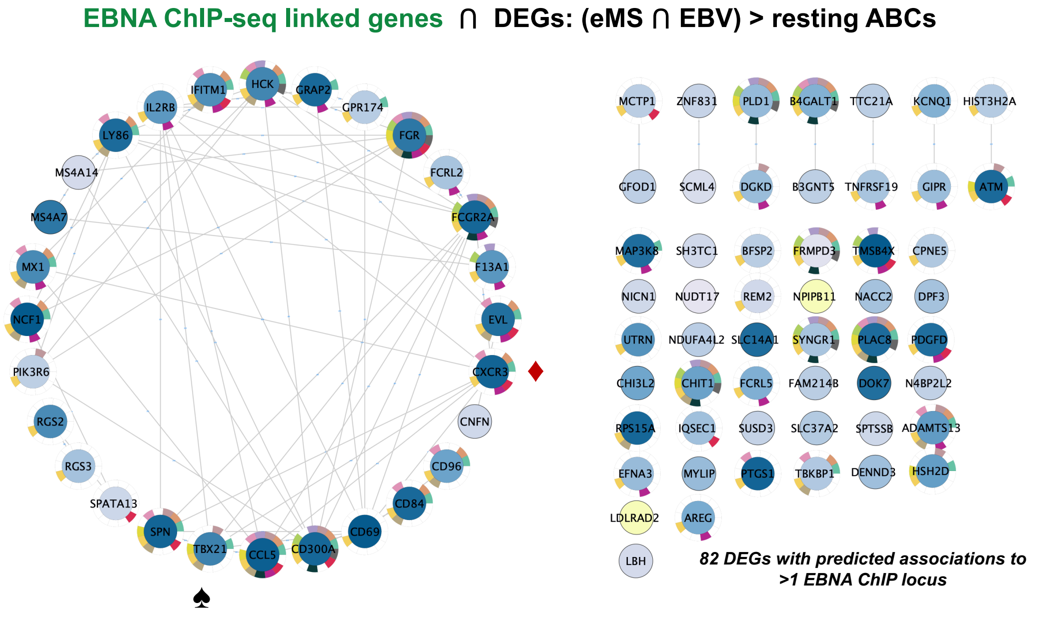

GO Term ID	GO Biological Process	FDR	genes	background
GO:0002376	Immune system process	0.0046	28	2481
GO:0006955	Immune response	0.0046	22	1588
GO:0001775	Cell activation	0.0076	17	1075
GO:0006887	Exocytosis	0.0442	13	789
GO:0006952	Defense response	0.0442	17	1296
GO:0045055	Regulated exocytosis	0.0442	12	697
GO:0045321	Leukocyte activation	0.0442	14	929
GO:0050896	Response to stimulus	0.0442	52	8046
GO:0051707	Response to other organism	0.0442	17	1256
GO:0042119	Neutrophil activation	0.0467	10	497
GO:0007166	Cell surface receptor signaling pathway	0.0468	23	2325
GO:0030334	Regulation of cell migration	0.0468	13	865
GO:0043299	Leukocyte degranulation	0.0468	10	506

B

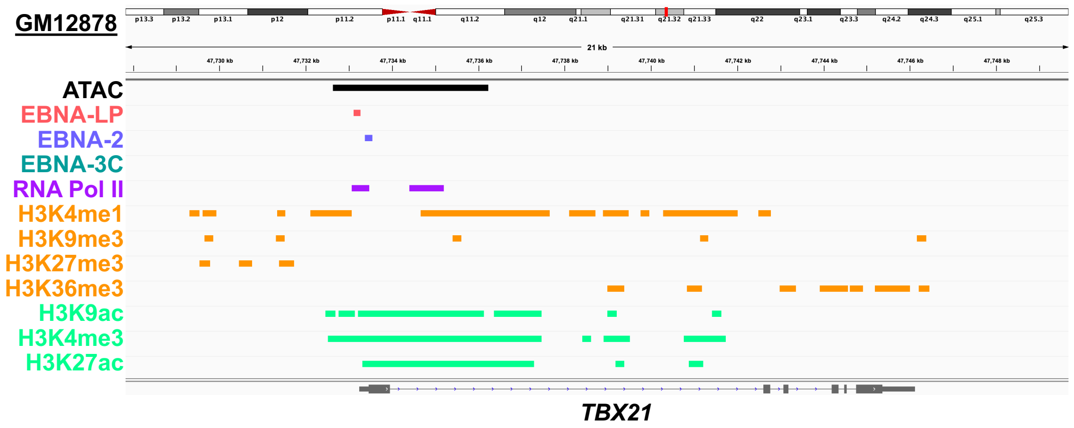

Figure S17. Genes expressed in ABCs from eMS and *de novo* EBV datasets with predicted *cis*-regulatory linkage to EBV nuclear antigen (EBNA) binding sites.

A) Gene network representation and ontology of EBNA binding site-associated genes with elevated expression in eMS and *de novo* EBV-infected versus resting ABCs. These EBNA-linked differentially expressed genes include *TBX21* (T-bet) and *CXCR3* (denoted by spade and diamond icons, respectively).

B) ATAC-seq and ChIP-seq data from the GM12878 cell line (EBV-transformed B cells) identifying open chromatin, EBNA binding sites, and epigenetic marks for active transcription at the *TBX21* transcription start site.

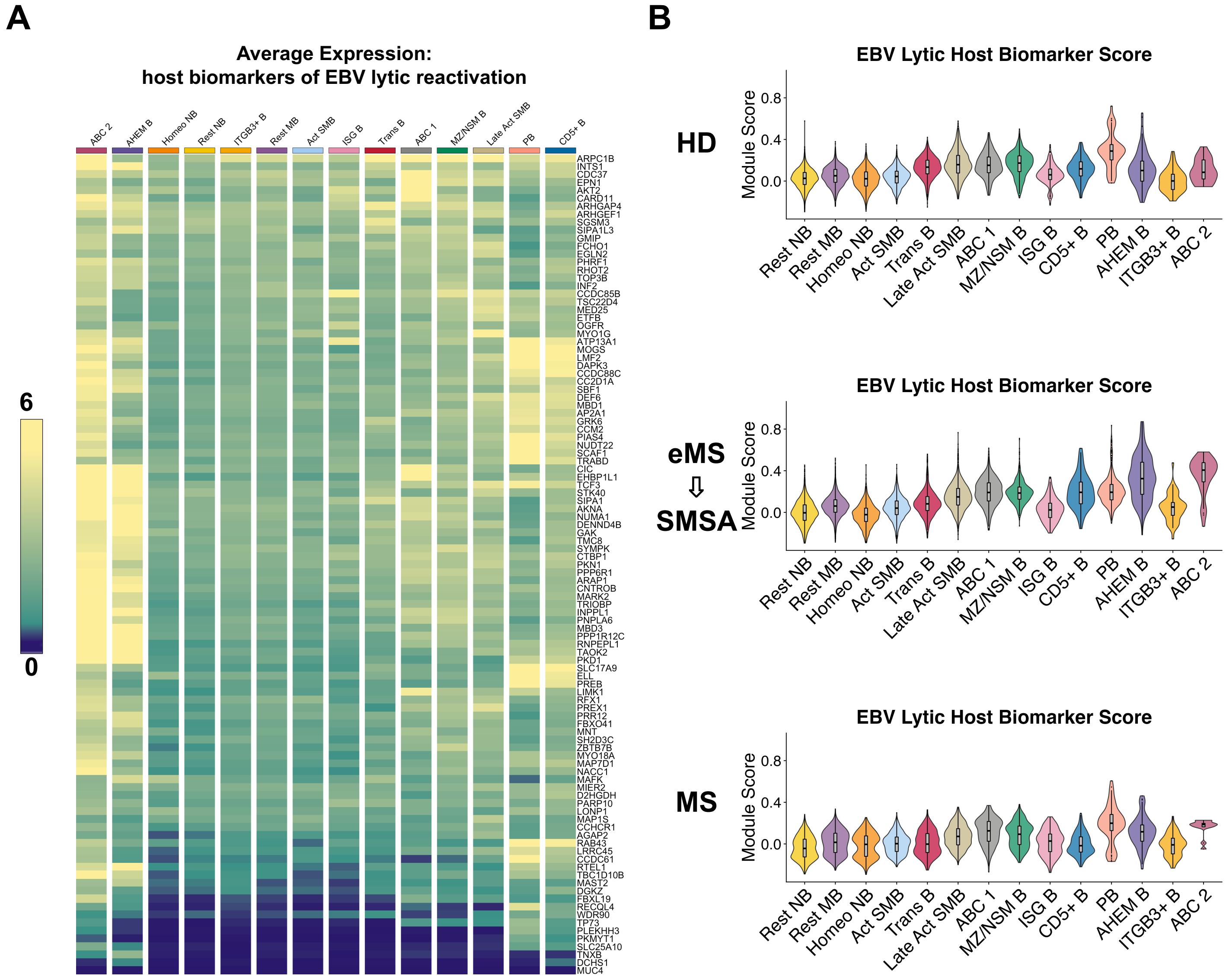

Figure S18. Expression of host cell biomarkers of EBV lytic reactivation from latency by B subset.
A) Average expression of top cellular mRNA biomarkers of EBV lytic reactivation (124) by annotated B subset.
B) Module scores for host biomarkers of EBV lytic reactivation in healthy donors (top row), people with eMS with subsequent MS activity (middle row), and people with MS (bottom row) by annotated B subset.

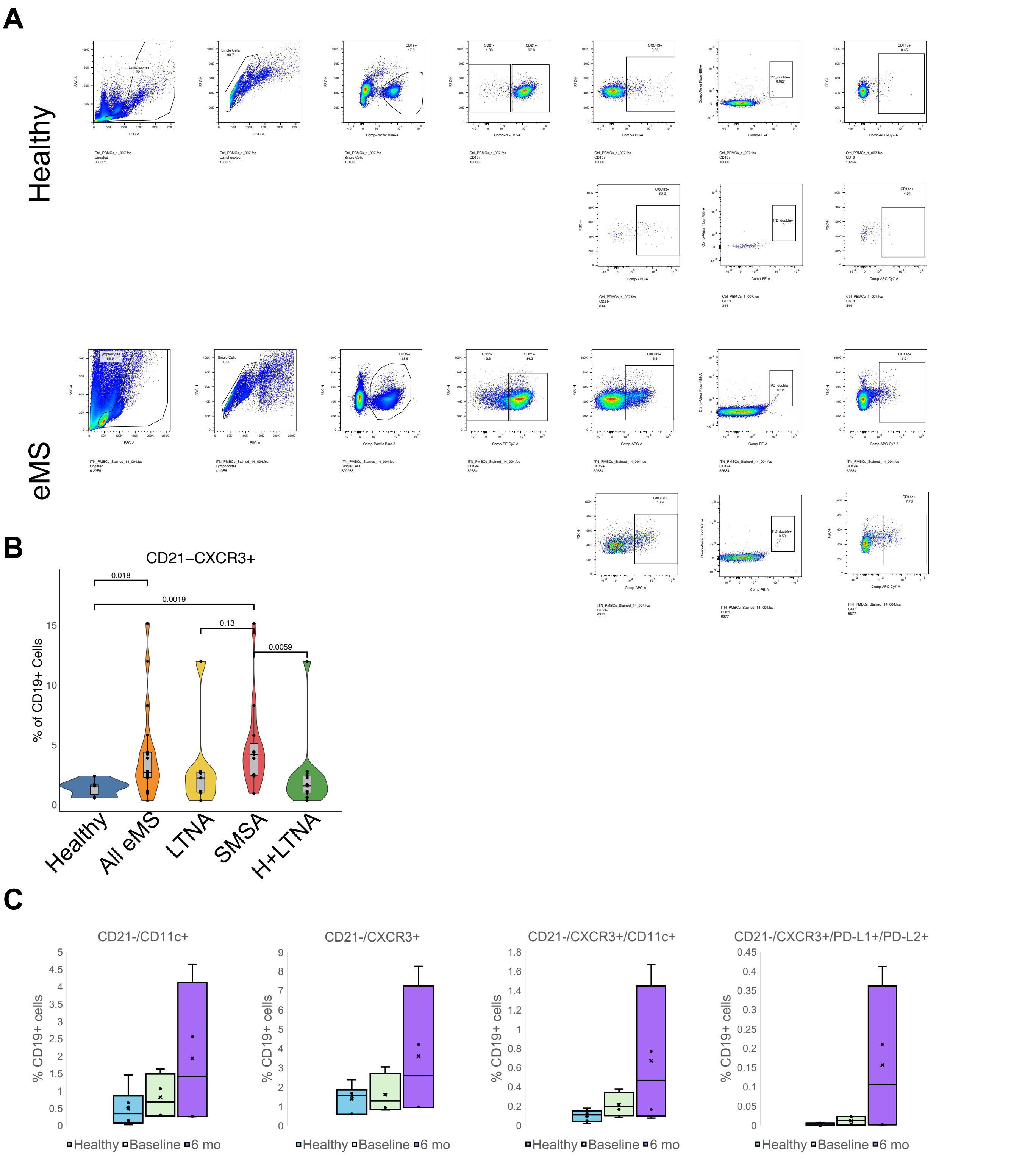

Figure S19. Gating strategy and longitudinal data for flow cytometry surface biomarkers

A) All gates for healthy control (top rows) and eMS (bottom rows) PBMC samples presented in Figure 5. Gates for all surface markers were set based on unstained and n-1 panel controls.

B) Quantification and statistical comparison of $CD19^{+}CD21^{lo}CXCR3^{+}$ cells between healthy (n=6), all eMS (n=18), eMS→LTNA (n=7), eMS→SMSA (n=11), and healthy + LTNA (n=13) groups. All statistics represent p values from Wilcoxon rank-sum tests (two-sided).

C) Surface expression in healthy controls (n=6) as well as eMS case-matched baseline and 6-month follow-up samples (n=4 individuals). All data are presented as the percent of $CD19^{+}$ cells.
